# Magnetic-Field-Assisted Fe Nanowire Conformable Aerogels Galvanically Displaced to Cu and Pt for Three-Dimensional Electrode Applications

**DOI:** 10.1021/acsami.5c00693

**Published:** 2025-04-28

**Authors:** Rosemary L. Calabro, Garret L. Longstaff, Edward M. Tang, Veronika M. Xiao, Alexa S. Zammit, Felita W. Zhang, Enoch A. Nagelli, Peter H. Chapman, Timothy J. Lawton, Mark A. Allen, Anchor R. Losch, Jesse L. Palmer, Alexander D. Ciampa, Ian Z. Burpeau, Veronica M. Lucian, Galen T. Mandes, Stephen F. Bartolucci, Joshua A. Maurer, F. John Burpo

**Affiliations:** † Department of Chemistry and Life Science, United States Military Academy, West Point, New York 10996 , United States; ‡ U.S. Army Combat Capabilities Development Command-Armaments Center, Watervliet Arsenal, New York 12189, United States; § Photonics Research Center, United States Military Academy, West Point, New York 10996 , United States; ∥ Department of Physics and Nuclear Engineering, United States Military Academy, West Point, New York 10996 , United States; ⊥ U.S. Army Combat Capabilities Development Command-Soldier Center, Natick, Massachusetts 01760, United States; # U.S. Army Combat Capabilities Development Command-Army Research Laboratory, Proving Ground, Aberdeen ,Maryland 21005, United States

**Keywords:** nanowires, nanotubes, aerogels, iron, platinum, copper, 3D electrodes, fuel cells

## Abstract

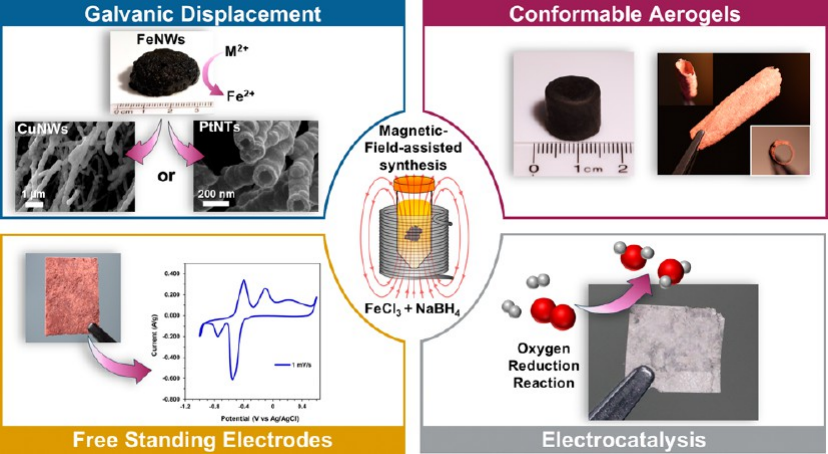

There is an increasing need for free-standing, conformal electrodes for practical energy storage devices. To address this, we demonstrate the magnetic-field-assisted synthesis of interpenetrating Fe nanowire (FeNW) gels without the use of templates or composite scaffold material over a range of magnetic fields. In either a wet gel or a supercritical dried state as an aerogel, the FeNWs may be pressed into thin or conformal films. Varying the applied magnetic field strength with a solenoid during chemical synthesis resulted in increased nanowire length and local orientation of the FeNWs with increasing magnetic field strength, with approximately 80 nm diameters across field strengths of 0–150 mT. Flowing K_2_PtCl_4_ or CuSO_4_·5H_2_O solutions through the wet iron gels to achieve the near complete galvanic displacement of iron to the more noble [PtCl_4_]^2–^ and Cu^2+^ ions resulted in either platinum nanotubes (PtNTs) or copper nanowires (CuNWs) while maintaining a percolating network structure. Similar to the FeNW gels, the PtNT and CuNW gels were able to be supercritical dried and/or pressed into thin or conformal electrode films. CuNW and PtNT films demonstrated good potential as capacitive and oxygen reduction reaction electrodes, respectively. The magnetic-field-assisted synthesis of ferromagnetic iron nanowires offers a simple, rapid, and tunable method that, when combined with galvanic displacement with more noble metal ions, may enable a wide range of metal, alloy, and multimetallic nanowires and nanotubes for energy storage, sensing, and catalytic applications.

## Introduction

The fast pace of technological development, with a vast array of device form factors and power requirements, has increased the need for synthesis and assembly strategies for inexpensive, lightweight electrodes that increase active materials loading and enable practical device integration.^1−3^ Nanowires for energy storage and sensor applications have been extensively used for their high surface area-to-volume ratios.^1^ Hierarchically assembled and percolating nanowire networks have also been widely used for flexible electronic devices.^4^ However, there are several challenges in designing and implementing nanowire electrodes. Performance of batteries, capacitors, supercapacitors, and fuel cells tends to be evaluated by mass-specific parameters rather than by accounting for the ancillary mass of current collectors, conductive additives, and organic binders. As a consequence, full system mass performance is often considerably lower^5^; this challenge is especially significant for 2-dimensional geometries. 3-dimensional (3D) electrodes have been proposed as a way to allow hierarchically designed materials with open pore networks. This maximizes the inclusion of active materials and provides conductive pathways throughout the full electrode thickness, thus maximizing both energy and power densities.^6^ Another challenge in electrode design is the need to accommodate irregular and confined geometries such as portable electronics, microelectromechanical systems (MEMS) devices, and military ordnance and equipment.^7^ Additionally, noble metals and transition metals are the basis for many of the catalytic and energy storage electrodes. Scarcity and the high cost of noble metals are driving the need for cheap transition metal solutions.^8^ Therefore, a synthesis method to achieve conformal, 3D hierarchically structured electrodes with tunable material phase composition would be highly beneficial for a wide range of energy storage, catalysis, and sensor applications.

3D electrodes have been achieved through various means including 3D nanowire assemblies,^9,10^ vertically aligned and patterned nanowires,^11^ anodic aluminum oxide (AAO) and inverse opal templates,^12^ and composite materials.^5,13,14^ Aerogels are an interesting class of 3D materials that possess many favorable properties that enable their application in numerous areas including 3D electrodes, energy storage,^13^ and electrocatalysis.^15^ They are derived from gels–which are nonfluid colloidal or polymer networks–and arise when the liquid of a gel is replaced with air.^15^ Aerogels have high surface areas that allow many accessible sites for catalysis or charge buildup, and they have high porosities, which allow flow of electrolytes and reactants to pervade the entire material. Meanwhile, they are lightweight with low densities typically ranging on the order of 0.01–0.5 g cm^–3^, and in some cases, they can be conformed into freestanding films without the use of additives or binders.^16^ While much consideration has been given to silica and polymer-based aerogels, noble and transition metal aerogels have recently shown promise for many electrode applications.^8,15,17−20^ Typically composed of nanoparticle building blocks, these metal aerogels can have tunable properties based on their elemental composition, surface environments, feature size of their building blocks, and inclusion of many additives or binders. For instance, modification of the nanoparticle capping ligand can modulate the valence state of the metals and thus tune the electrocatalytic performance.^21^ Bimetallics can allow improvement of electrocatalytic performance through the tuning of the metal ratios.^22^ Meanwhile, hybrid materials have been shown to enhance both electrochemical and mechanical properties.^13^

Typical strategies to prepare metal aerogels include sol–gel synthesis,^20^ destabilization of metal nanoparticle solutions,^21,22^ microwave synthesis,^23^ hydrothermal reactions,^13^ and templated synthesis.^24^ However, these methods are often time-consuming and difficult to scale while yielding aerogels that are mechanically fragile due to limited ligament length^17^ or have catalytic sites blocked by additives, binders, or surfactants,^21^ and efforts have been made to prepare ligand-free or undercoordinated metal aerogels.^21,25^ Reports of chemical reduction from high salt concentrations show the direct and rapid formation of metal aerogels, which allows scalability and accessible surfaces.^26−28^ Meanwhile, nanowire aerogels have been shown to possess good mechanical properties by featuring intertwined networks of high aspect ratio nanowires.^20,29^ A magnetic field-assisted synthesis (MFA) has been developed as a strategy to achieve 3D nanowire assemblies by using a magnetic field to direct nucleation and growth as ferromagnetic iron, cobalt, and nickel ions are chemically reduced.^30,19,31^ As ferromagnetic precursors are chemically reduced, they form nanoparticles. The magnetic field promotes alignment of these nanoparticles into nanochains, which then coalesce together to form nanowires,^32−34^ and the magnetic field strength can be used to tune the feature size and aspect ratio of the formed structures.^34−36^ MFA synthesis with applied magnetic fields over a range of 0–1.4 T^30^ has been applied to produce a variety of magnetic nanowire structures including iron oxide nanowires with tunable aspect ratios based on pH,^37^ cobalt nanowires 20–50 nm in diameter with aspect ratios over 1000^38^; nickel nanowires^39^ and microwires,^40^ cobalt nanowire aerogels with tunable lengths and aspect ratios based on applied magnetic field strength,^16^ iron–nickel alloys through a coreduction,^41^ and iron–cobalt nanowires.^42^ However, many of these studies used hydrothermal conditions,^35,43^ required inert atmospheres,^42^ yielded mixed morphologies,^43^ or did not result in bulk 3D structures.^33,36^ Moreover, while MFA synthesis allows the formation of a variety of anisotropic nanostructures, it is limited in scope to ferromagnetic materials.

Galvanic displacement, however, has been established as a synthetic strategy that allows for the electrochemical exchange of one metal with another through a spontaneous reaction based on their reduction potentials,^44^ and this technique has been used to prepare various metal aerogels and nanowires.^45−47^ With galvanic displacement, metals that have a lower reduction potential can serve as a sacrificial template, which reduce and are then displaced by metal ions in solution that have a higher reduction potential.^48,49^ The displacing metal is then deposited on the nanostructure surface, while the template metal is oxidized and dissolves away into solution. This typically lends to the formation of hollow, cage, or tube-like structures and if enough displacing metal is provided, can allow complete replacement of the sacrificial template.^44,49^ While Cu and Pt are not ferromagnetic, and thus cannot be used for MFA, they have higher reduction potentials than iron.^49^ This allows iron nanowires (FeNWs) produced through MFA to serve as a sacrificial template and be replaced with the more noble Cu or Pt ions, which are important metals for applications in energy storage and electrocatalysis.^1,15^

In this study, we demonstrate the MFA synthesis of interpenetrating FeNW gels without the use of templates or composite scaffold material from relatively high reagent concentrations over a range of magnetic fields. In either a wet gel or a supercritical dried state as an aerogel, these FeNWs may be pressed into thin or conformal films. Varying the applied magnetic field strength from 0 to 150 mT with a solenoid resulted in an increased nanowire length and local orientation of the FeNWs with increasing magnetic field strength. Flowing K_2_PtCl_4_ or CuSO_4_·5H_2_O solutions through the wet FeNW gels allows near complete galvanic displacement of iron to the more noble [PtCl_4_]^2–^ and Cu^2+^ ions and resulting in either platinum nanotubes (PtNTs) or copper nanowires (CuNWs) that maintain the percolating network structure. Like the FeNW gels, the PtNT and CuNW gels could also be supercritical dried and/or pressed into thin or conformal electrode films. Cu nanowire and Pt nanotube films were demonstrated as capacitive and oxygen reduction reaction electrodes, respectively. The MFA synthesis of ferromagnetic iron nanowires offers a simple, rapid, and tunable synthesis method and can be combined with galvanic displacement by more noble metal ions. MFA synthesis of Fe nanowire aerogels and films offers a platform to achieve a wide range of metal, alloy, and multimetallic nanowires and nanotubes for energy storage, sensing, and catalytic applications.

## Experimental Methods

### Materials

Iron(III) chloride hexahydrate (reagent grade, ≥98%), copper(II) sulfate pentahydrate (ACS reagent, ≥98.0%), potassium tetrachloroplatinate (II) (98%), and sodium borohydride (≥98.0%) were obtained from Sigma-Aldrich and used without any further purification.

### Synthesis

All synthesis and characterization methods are explained in detail in the Supporting Information. In summary, a custom-made solenoid was used for chemical reduction of 0.1 M FeCl_3_·6H_2_O with 0.1 M NaBH_4_.^16^ Reagents were mixed within the solenoid across a range of magnetic field strengths of 0, 9, 19, 37, 75, and 150 mT and were allowed to chemically reduce for 2 min, followed by rinsing the resulting metal gels in deionized water for 1 h (Figure S1). Aerogels with additional reagent concentrations ranging from 0.025 to 0.075 M FeCl_3_·6H_2_O were also synthesized (Figure S2). Drying was performed either by gently pressing the wet gels between two Teflon plates and then wicking dry, or by solvent exchange with ethanol and supercritical drying with a Leica EM CPD300 Critical Point Dryer (Leica Microsystems, Deerfield, IL, USA). Galvanically displaced nanowires and nanotubes were formed by flowing 0.5 M CuSO_4_·5H_2_O or 0.1 M K_2_PtCl_4_ through wet iron nanowire gels, respectively. The galvanic displacement flow apparatus using a vacuum pressure-driven flow is shown and described in Figure S3. The resulting Cu nanowire or Pt nanotube gels were either gently pressed into a thin film or solvent exchanged with ethanol and supercritical dried.

### Material Characterization

A JEOL IT500HR scanning electron microscope (SEM) (JEOL USA, Peabody, MA, USA) was used to collect micrographs and energy-dispersive X-ray (EDS) spectra. ImageJ was used to perform analysis of the nanowire structure in SEM micrographs.^50^ Orientation Order Parameter (OOP) was quantified from SEM images to compare nanowire alignment between magnetic field strengths using MATLAB code GTFiber.^51^ X-ray diffraction (XRD) analysis was performed with a PanAlytical Empyrean diffractometer in a SAX/WAXS configuration using 45 kV and 40 mA. Transmission electron microscopy (TEM) was collected with a JEOL-2100F TEM system (JEOL USA, Peabody, MA) with a Gatan Oneview CCD camera. Selected area electron diffraction (SAED) was performed with an 80 cm camera length, and electron diffraction patterns were compared with data from The Materials Project.^52^ X-ray photoelectron spectroscopy (XPS) spectra were collected with a K-Alpha XPS System (Thermo Fisher Scientific, Waltham, MA, USA) by using a monochromated Al K_α_ X-ray source. Nitrogen adsorption–desorption isotherms were measured with a Micrometrics ASAP 2000 Plus porosimeter (Micrometrics, Norcross, GA, USA). Vibrating sample magnetometry (VSM) was collected with a MicroSense EZ7 vibrating sample magnetometer (MicroSense, Lowell, MA, USA). Inductively coupled plasma optical emission spectroscopy (ICP-OES) was measured using a PerkinElmer Avio 500 ICP Optical Emission Spectrometer (PerkinElmer, Shelton, CT, USA). FeNW aerogel stress–strain curves were collected using a TA Instruments Discovery HR-3 rheometer (TA Instruments, New Castle, DE, USA) using 40 mm parallel plates at a strain rate of 50 μm/s for a strain range of 0–0.6 mm/mm.

### Electrochemical Analysis

All electrochemical analyses were performed with a Biologic VMP-3 (Biologic-USA, Knoxville, TN, USA) potentiostat using a 3-electrode cell. Ag/AgCl reference electrodes were used with a platinum wire counter electrode in either H_2_SO_4_ or KOH electrolytes. The working electrode was a lacquer-coated platinum wire with an exposed tip in contact with the sample film. Rotating disk electrode (RDE) experiments were conducted with a Pine Research Instrumentation Modulated Speed Rotator (Model AFMSRCE, Pine Research Instrumentation, Durham, NC, USA) at 400, 800, 1200, and 1600 rpm with pressed film sample material cast on glassy carbon electrodes using a 5% stock Nafion solution (Sigma-Aldrich). A control electrode of 5 μL of 5% stock Nafion was cast on a glassy carbon electrode, and was used with a 0.5 M H_2_SO_4_ electrolyte and the same rotation rates. Electrochemical impedance spectroscopy (EIS) spectra were collected over a range of 1 MHz to 1 mHz scan frequencies in galvanostatic mode with 500 μA peak current. The Z-fit function in EC-Lab, v.11.10 (Biologic), was used for resistor-inductor-capacitor (RLC) model fitting of EIS spectra. See the Supporting Information for fitting details.

## Results and Discussion

**Figure 1:**
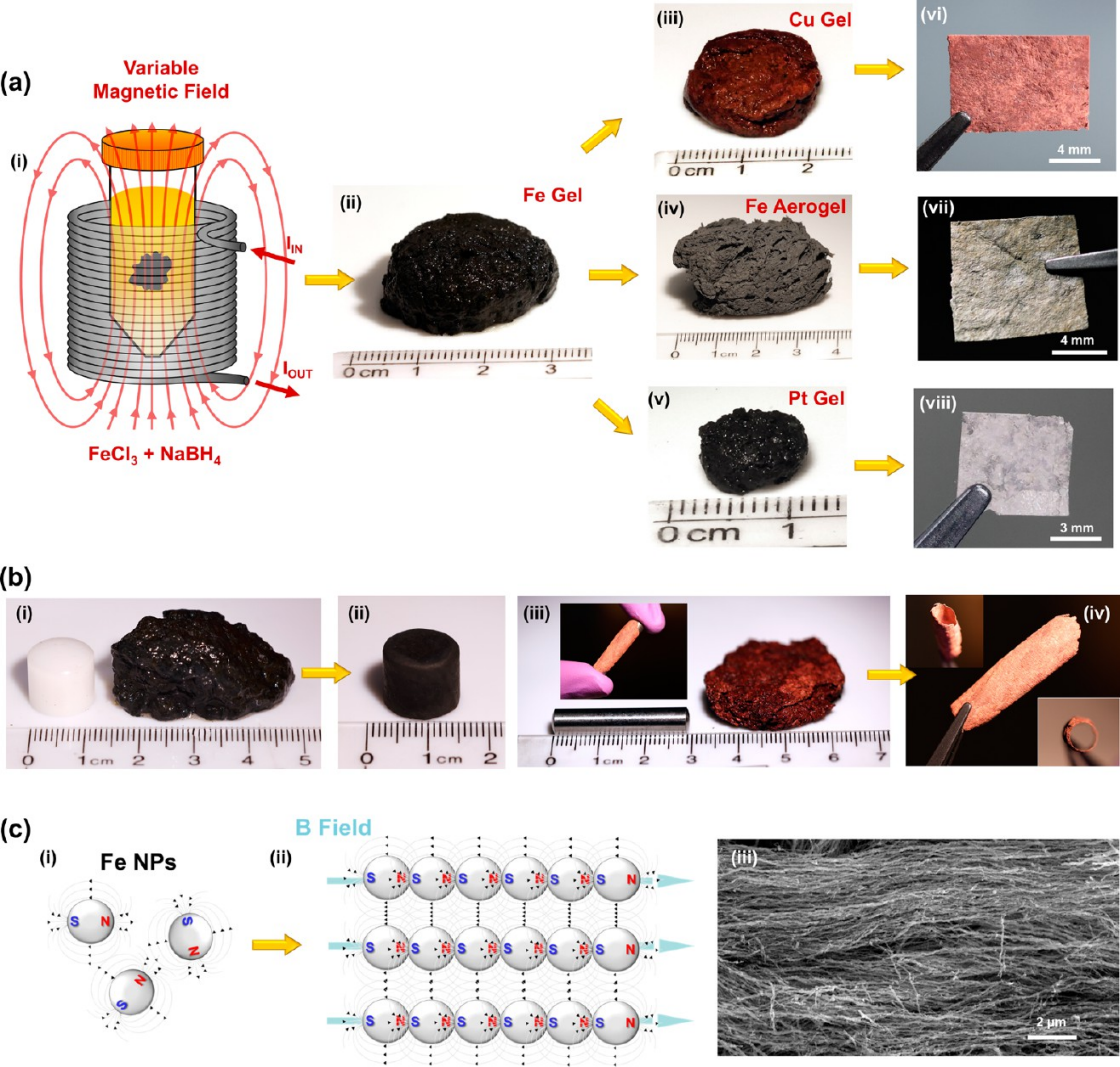
MFA synthesis scheme (a): FeCl_3_·6H_2_O and NaBH_4_ are mixed inside a variable magnetic field strength solenoid (i) to form a metal gel (ii); the metal can be solvent exchanged and supercritical dried to form an Fe aerogel (iv), galvanically displaced in CuSO_4_·5H_2_O to form a Cu metal gel (iii), or galvanically displaced in K_2_PtCl_4_ to form a Pt metal gel (v). Metal gels or supercritical dried aerogels can be pressed into free-standing films (vi–viii). Aerogel conformal coatings (b) of an iron gel conformed on a Teflon stub (i–ii); a copper gel conformed around a stainless-steel cylinder (iii); gel from (iii) with the stainless-steel cylinder removed (iv). Proposed mechanism (c): initial Fe nanoparticle formation (i); nanoparticle alignment and coalescence along magnetic field lines (ii); and extension of coalesced nanoparticles into nanowires (iii).

A custom solenoid was used to apply a range of magnetic field strengths during the chemical reduction of 0.1 M FeCl_3_·6H_2_O with 0.1 M NaBH_4_, as shown in Figure 1a. A fixed magnet was used for 150 mT field strengths (Figure S1). The initial chemical reduction resulted in a rapid solution color change to black, along with a vigorously bubbling solution due to the evolution of hydrogen gas as a reaction side product. After a few seconds of the reaction, a black gel could be observed near the top of the reaction vessel. The reaction was allowed to proceed until gas evolution ceased, forming a wet iron metal gel, as shown in Figure 1a(ii). After rinsing with deionized water, solutions of either 0.5 M CuSO_4_·5H_2_O or 0.1 M K_2_PtCl_4_ were flowed through the iron gels to facilitate galvanic displacement of the iron to copper and platinum gels, respectively (see Figure S3 for a scheme of the flow apparatus). In the discussion that follows, nanowire (NW) and nanotube (NT) aerogel and film samples are designated as follows: iron–FeNW, copper–CuNW, and platinum–PtNT. Fe, Cu, and Pt metal gels could all be gently pressed into thin films and ambiently dried as shown in Figure 1a(vi, viii). Alternatively, after rinsing the wet gels with water, a solvent exchange with ethanol, followed by supercritical drying, resulted in iron aerogels as seen in Figure 1a(iv). Like the wet metal gels, supercritical dried aerogels could also be pressed between two plates into thin films, as shown in Figure 1a(vii). To further demonstrate the versatility of this synthesis method, wet gels were conformally applied to various geometries, as shown in Figure 1b. An iron gel was conformally pressed around a Teflon stub shown in Figure 1b(i–ii), and a copper gel was wrapped around a stainless-steel cylinder, which could then be detached from the cylinder and maintain a free-standing tube structure shown in Figure 1b(iv). This demonstrates the ability of these aerogels to access various form factors according to the requirements of the intended application, while retaining their porous structure. The aerogels can be left as a monolithic aerogel or can be conformed into various shapes at different points in the processing steps.

**Figure 2:**
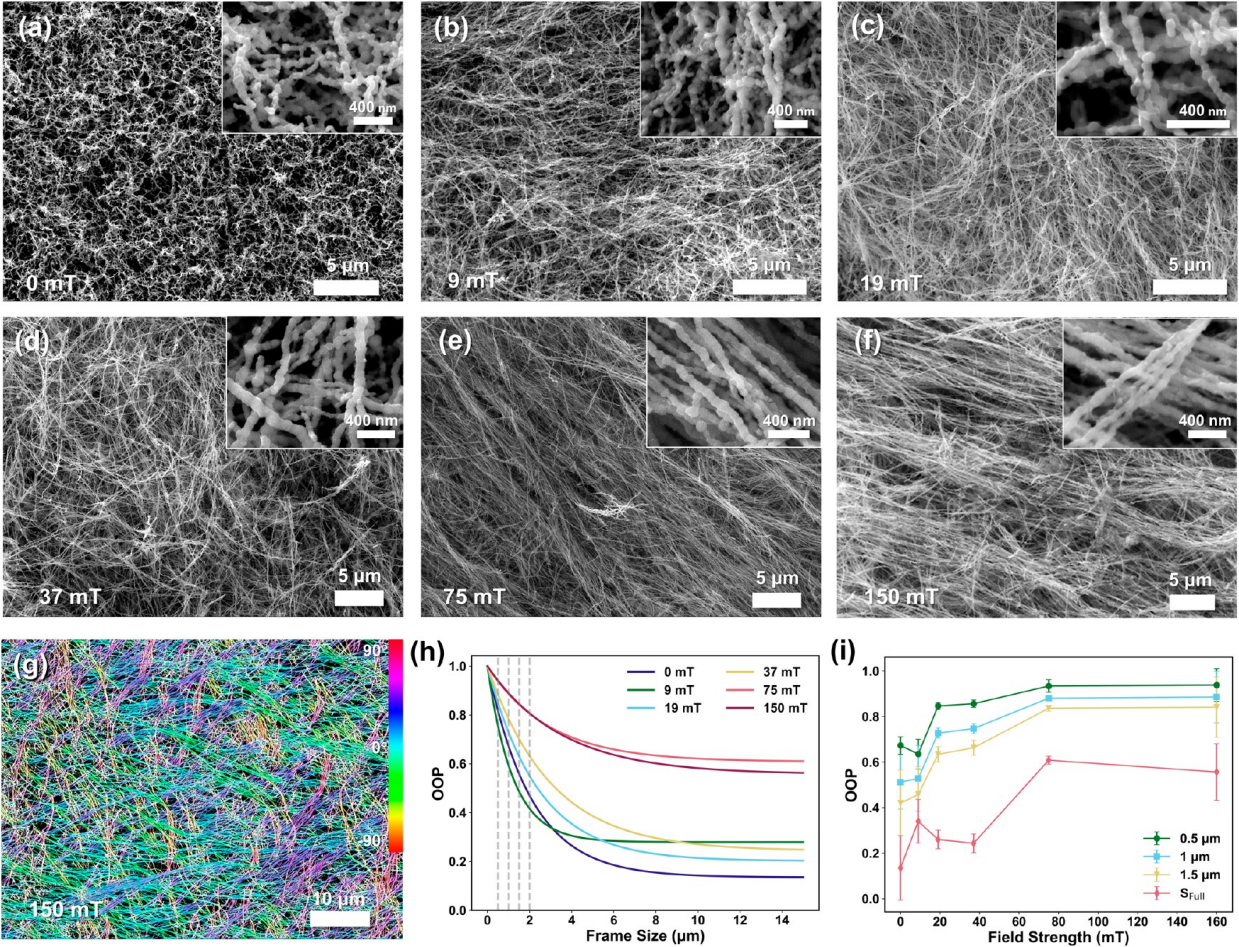
Scanning electron micrographs of iron nanowires synthesized in magnetic fields with strengths of 0 mT (a), 9 mT (b), 19 mT (c), 37 mT (d), 75 mT (e), and 150 mT (f). Colorized nanowire angular orientation of FeNW synthesized in a 150 mT field (g). Orientation order parameter (OOP) versus image frame size based on colorized nanowire angular orientation (h). Average OOP versus synthesis field strength (i).

The chemical reduction reaction is evidenced by a quick solution color change to black, followed by the development of a metal gel, and SEM imaging of pressed aerogel films reveals long nanowires of coalesced nanoparticles. These observations suggest the mechanism depicted in Figure 1c. Similar to other magnetic-field-assisted nanowire studies,^40,53,54^ the proposed mechanism begins with the initial formation of iron nanoparticle clusters (Figure 1c(i)), which are magnetically responsive and align along the externally imposed field lines (Figure 1c(ii)). Continued chemical reduction of iron ions and free energy minimization of nanoparticles result in coalescence into locally aligned nanowires with the beads-on-a-string appearance seen in the SEM micrograph in Figure 1c(iii). EDS confirmed that the FeNWs were 77.3 atomic percent iron (Table 1, Figure S4), with oxygen accounting for the remaining atomic percentage. The mechanism scheme suggests a hypothesis that nanowire length and local relative ordering of nanowires might be controlled relative to the imposed magnetic field strength during synthesis. To test this hypothesis, iron nanowire synthesis was conducted at field strengths of 0, 9, 19, 37, 75, and 150 mT, with the resulting scanning electron micrographs for each shown in Figure 2a–f. In the absence of a magnetic field, chemical reduction of iron ions resulted in a 3-dimensional network of intertwined and interconnected nanowires with segment lengths on the order of 100 s of nanometers. As the applied magnetic field was increased up to 150 mT, the nanowire lengths increased to 10 s of micrometers for over a 2-order of magnitude change for a relatively small applied field range. The exact determination of nanowire length is difficult due to the over- and under-lapping nature of the nanowires in both the aerogel and pressed film samples. However, previous studies showed that increasing the applied field strength can increase the length, aspect ratio, and yield of nanowires within a sample.^16,43^ This is most notable when comparing the samples prepared under zero field conditions to those prepared at 150 mT. While the particles prepared at zero field (Figure 2a) have some anisotropy, applying an increasing field strength resulted in significantly longer wires (Figure 2b–f). Changing the reagent concentrations could also impact the FeNWs (Figure S2), with lower reagent concentrations allowing less densely packed nanowires.

**Table 1:** Summary Table of Measurement Parameters for the FeNWs, CuNWs, and PtNTs Synthesized in a 150 mT Magnetic Field Obtained from SEM Image Analysis, XRD Scherrer Analysis, EDS, ICP-OES, XPS, Nitrogen Adsorption–Desorption, and Specific Capacitance (*C*_sp_)

**measurement parameter**	**FeNW**	**CuNW**	**PtNT**
nanowire diameter (nm)	95 ± 24	228 ± 57	139 ± 20 (outer diameter), 58 ± 12 (inner diameter)
crystallite size (nm)	17.1	30.8	7.7
EDS atomic percentages (%)	77.3 Fe	99.6 Cu, 0.4 Fe	97.8 Pt, 2.2 Fe
ICP atomic percentages (%)^1^	77 ± 4 Fe	95.6 ± 0.4 Cu, 4.4 ± 0.4 Fe	93.0 ± 1.0 Pt, 7.0 ± 1.0 Fe
XPS atomic percentages (%)	19.64 Fe	24.89 Cu	46.45 Pt, 8.45 Fe
BET surface area (m^2^ g ^–1^)	54.1	5.4	5.8
BJH pore volume (cm^3^ g^–1^)	0.163	0.024	0.011
BJH pore diameter d*V*(*d*) (nm)	7.4	17.1	18.9
*C*_sp_ (F/g) from EIS	NA	5.4	4.1

1 Calculated assuming oxygen accounts for the remaining weight percent.

To determine the influence of the applied magnetic field during synthesis on local nanowire orientation and ordering, the relative nanowire angular orientation was colorized and then image processed based on a common orientation vector as seen in Figure 2g. The colorized nanowires revealed numerous lengthwise regions with roughly parallel nanowires shown in the same color. Following these parallel nanowires along their longitudinal direction revealed bending deviations from a straight-line path. To quantify the degree of relative nanowire ordering resulting from the applied field strength, orientation order parameter (OOP) analysis was performed on SEM images, with image preprocessing steps resulting in a colorized orientation map of skeletonized nanowires, with colors representing absolute fiber orientation (see the Supporting Information for a full description of OOP image processing and analysis). This map was subsequently used to quantify OOP. An OOP value of 1 indicates parallel ordering of linear elements, while an OOP value of 0 indicates completely random nanowire orientation, with intermediate values resulting from varied degrees of relative ordering to a common vector.^51^ Each SEM image was sampled with increasing frame sizes to estimate local-to-global degrees of relative orientation between the nanowires. As seen for representative samples in Figure 2h, at analysis frame sizes much less than 1 μm, OOP values approached unity for all field strengths. As the frame size was increased, the number of OOP values decreased as more of the image sample was included in the analysis. Once the analysis frame size exceeded approximately 6 μm, global OOP values were approached asymptotically (see Table S1 for OOP values as a function of synthesis magnetic field strength and analysis frame size). A few trends in the OOP analysis have emerged. First, in general, the OOP values increased with increasing applied magnetic fields during chemical reduction of iron nanowires. The global OOP value for the iron gels synthesized in the absence of a magnetic field was the lowest average global OOP value of 0.14 ± 0.14, while at the maximum applied magnetic field of 150 mT, the OOP value was 0.56 ± 0.12. However, FeNW films synthesized in a 75 mT field had the highest OOP of 0.61 ± 0.02, and the OOP values were not consistently progressive with the magnetic field at intermediate values, as nanowires synthesized in a 9 mT field had a higher OOP than those synthesized in 19 and 37 mT fields. To examine the sensitivity of OOP to the analysis frame size, Figure 2i plots OOP values as a function of the applied magnetic fields for analysis frame sizes of 0 μm, 1 μm, 1.5 μm, and “*S*_Full_” when the OOP approaches a global constant value. The same general increase in the OOP with increasing magnetic field strength is seen across each level of “zoom,” or analysis frame size. However, at the lower field strengths between 9 and 37 mT, OOP values do not monotonically increase. There are a few synthesis and sample preparation conditions that likely contributed to lower magnetic field values, not consistently increasing the number of OOP values with increasing field strength. The first factor is likely the vigorous hydrogen gas evolution during chemical reduction (see Video S1). The consistent stream of large-scale gas bubbles relative to the feature size of the nascent iron nanoparticles likely disrupted their alignment and coalescence along weaker field lines. As nanowires formed and extended, crowding effects within the metal gel, combined with the intrinsic magnetic field of the iron nanowires themselves, may have contributed to the nanowire bending seen in SEM images in Figure 2. The overall trends of increasing nanowire length and local-to-global nanowire ordering suggest that even higher imposed magnetic field strengths during ferromagnetic nanowire synthesis might achieve more distinctly anisotropic materials.

**Figure 3:**
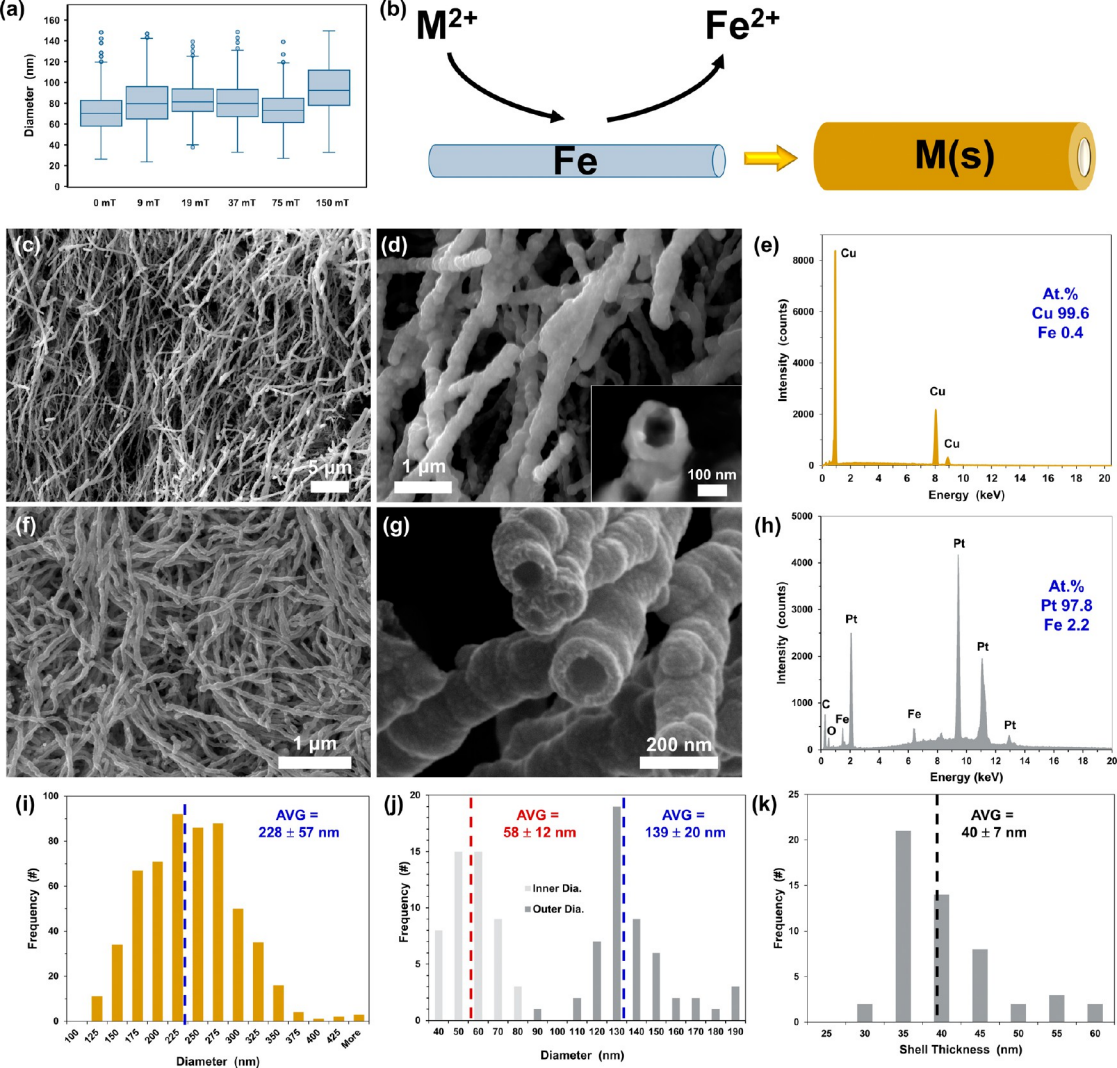
Iron nanowire diameters as a function of magnetic field strength during synthesis (a). Galvanic displacement scheme for iron nanowires as a sacrificial template (b). SEM images of Fe nanowires galvanically displaced to Cu nanowires (c, d). Energy dispersive X-ray spectroscopy (EDS) for copper nanotubes (e). SEM images of Fe nanowires galvanically displaced to Pt nanotubes (f, g). EDS spectrum for Pt nanotubes (h). Cu nanowire outer diameter distribution (i). Pt nanotube inner and outer diameter distributions (j). Pt nanotube shell thickness distribution (k).

The iron nanowires had diameters shown in Figure 3a ranging from 71 ± 18 nm prepared with zero magnetic field up to 95 ± 24 nm for 150 mT field strength. Nanowire diameters for intermediate magnetic field strengths between 9 and 75 mT ranged between 73 to 83 nm, all with overlapping standard deviation ranges (see Table S2 for values). The relative consistency of fiber diameters between field strengths is likely due to the rapid chemical reduction and nanoparticle coalescence kinetics, irrespective of field strength, and the increasing nanowire length with magnetic field strength suggests a direct relationship between the nanowire aspect ratio and field strength. Given the relatively low reduction potential of iron of −0.447 V vs SHE (for 2 electron oxidation, Fe → Fe^2+^ + 2e^–^), the iron nanowire gels offer the potential to serve as sacrificial templates via galvanic displacement for more noble metals, thereby offering a common 3-dimensional synthesis platform for a broad range of materials (Figure 3b). To demonstrate this, the abundant transition metal copper and the rare earth element platinum were selected to demonstrate galvanic displacement with FeNWs synthesized with a 150 mT magnetic field. A summary of the measured properties of the FeNWs, CuNWs, and PtNTs is presented in Table 1. As seen in Figure 3c,d, the displacement of iron with copper resulted in copper wires with an average diameter of 228 ± 57 nm (Figure 3i) and 99.6 atomic percent copper content based on EDS (Figure 3e). ICP-OES measurements (Figure S5, Tables S3 and S4) further confirmed that during galvanic displacement, a majority of the material is comprised of the displacing metal with minimal (<5 at. %) iron remaining. Cross-sectional images of the copper displacements revealed a hollow interior, indicating that the original iron nanowires are sacrificially dissolved when the Fe atoms yield their electrons to the Cu^2+^ ions as seen in Figure 3d(inset); however, a majority of the CuNWs were capped at the ends presenting nanowire rather than nanotube structure (Figure S6). When a platinum solution was flowed through the Fe metal gels, the galvanic displacement resulted in Pt nanotubes with a distinct inner boundary as seen in Figures 3g and S7–S8. Like with copper, EDS and ICP confirmed nearly complete exchange of Fe with Pt (Figure 3h, Table 1). The inner and outer nanotube metal shell diameter distributions are 58 ± 12 and 139 ± 20 nm, respectively (Figure 3j). The difference between the average outer and inner Pt nanotube diameters is 81 ± 7 nm, which corresponds to a metal shell thickness of 40 ± 7 nm (Figures 3k, S7–S8).

To assess the galvanic displacement mechanism of iron nanowires by copper and platinum ions and to predict the thickness of a resulting nanotube, a geometric model was used, assuming idealized nanowire and nanotube geometries, metal densities, and a complete electron displacement efficiency (Table S5). The resulting mass of Cu(s) and Pt(s) was assumed to form a nanotube with inner diameters of 95 and 58 nm, respectively, based on image analysis shown in Figure 3. The calculated outer diameters for Cu and Pt nanotubes are 134 and 144 nm, respectively. The geometrically predicted Cu outer diameter is less than the measured average outer diameter of 228 nm shown in Figure 3i. The larger observed Cu outer diameter is attributed to a rapid galvanic displacement of Fe by copper ions, resulting in dissolution of Fe nanowires, transferring electrons to Cu^2+^ along shorter segment lengths of the Fe nanowires. These shorter segment lengths and rounded end-caps of Cu nanotubes are seen in Figures 3c,d and S6. The geometrically predicted Pt nanotube outer diameter of 144 nm is close to the observed average outer diameter of 139 nm. In the case of Pt nanotubes, there is a predominantly outward galvanic displacement growth mechanism from the original Fe nanowire; however, the observed inner diameter in Figure 3j is less than the average Fe nanowire diameter of 95 nm, suggesting a partial inward growth mechanism. This process can be further expanded to other metals that have reduction potentials higher than those of iron, such as gold (Figure S9), thus demonstrating the versatility in using MFA nanowire synthesis coupled with galvanic displacement to achieve 3D nanowire aerogels and films.

**Figure 4:**
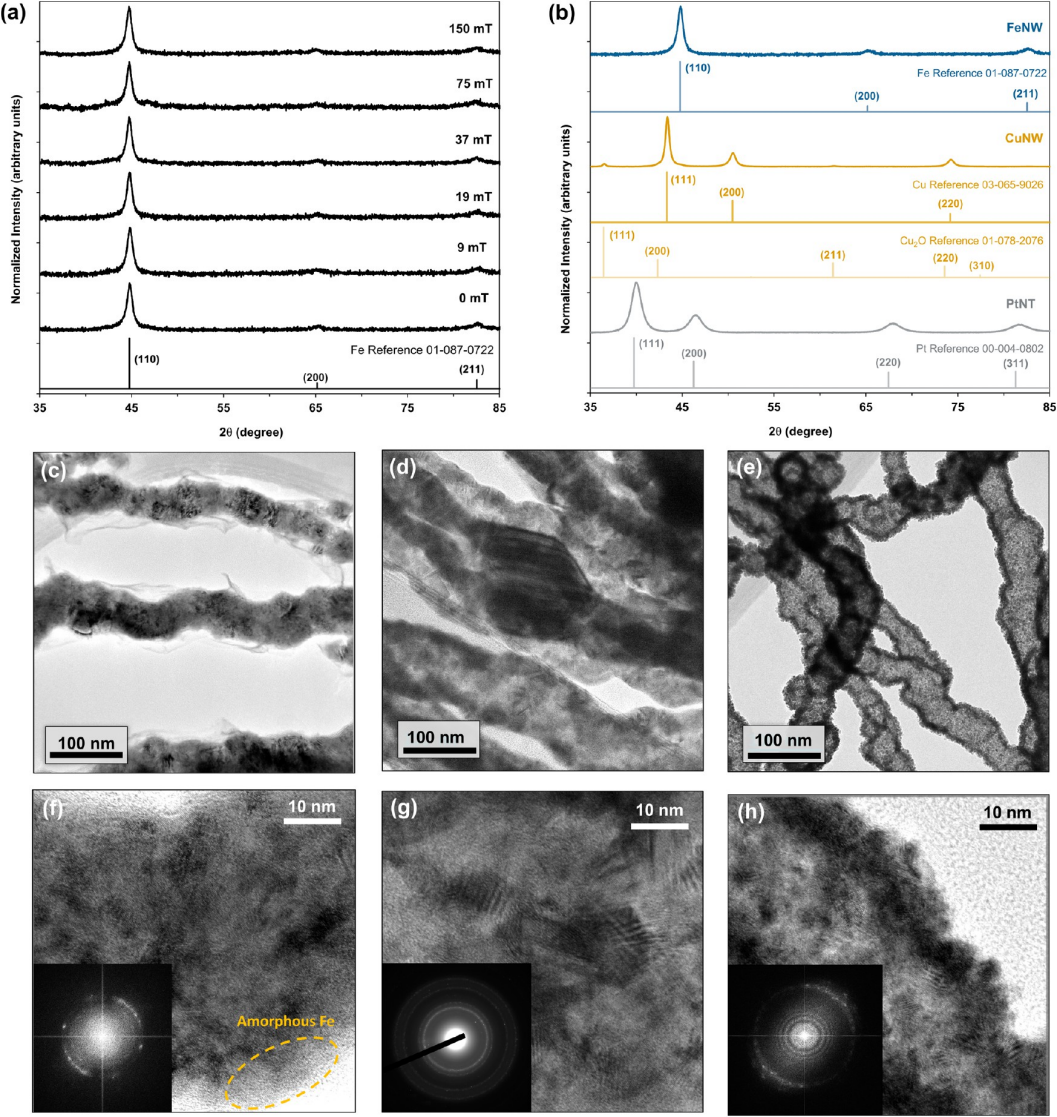
XRD patterns for the FeNWs synthesized at various applied magnetic field strengths (a) and for the FeNWs, CuNWs, and PtNTs (b) along with Fe, Cu, Cu_2_O and Pt reference patterns. Transmission electron microscopy images of the FeNWs (c, f), CuNWs (d, g), and PtNTs (e, h) collected at 100 kx (center row) and 800 kx (bottom row). The insets show electron diffraction patterns determined through Fast Fourier Transform (f, h) and SAED (g).

Figure 4a shows the XRD patterns for the FeNWs prepared at various magnetic field strengths. Regardless of the applied field strength used during synthesis, the FeNWs show the characteristic peaks for metallic cubic Fe. The average full width at half-maximum (FWHM) of the Fe(110) peak was consistent across all the field strengths indicating that the applied magnetic field during synthesis did not impact the crystallite size, and the average crystallite size according to the Scherrer equation was 17.5 ± 0.9 nm (Table S6) which is smaller than the measured feature size from image analysis of the FeNW diameters and widths. This suggests that the FeNWs are polycrystalline in nature, which can influence the electrical conductivity and magnetic properties^55^ of the material.

The XRD patterns for the FeNWs prepared with 150 mT and the PtNTs and CuNWs following galvanic displacement are presented in Figure 4b. Before galvanic displacement, the FeNWs had a similar pattern consistent with metallic cubic Fe and a crystallite size of 17.1 nm according to Scherrer analysis of the Fe(110) peak. Following galvanic displacement with Pt, the Fe peaks were entirely replaced with peaks characteristic of cubic Pt, which suggests complete replacement of the Fe with Pt and is consistent with the EDS and ICP-OES measurements. The PtNT peaks were broader than those of the FeNWs, which suggests a smaller crystallite size, and Scherrer analysis of the Pt(111) peak indicates an average crystallite size of 7.7 nm. Galvanic displacement of the FeNWs with Cu, however, resulted in narrower peaks characteristic of metallic cubic Cu and cubic Cu_2_O. The sample consisted of 92% metallic Cu, while the remaining 8% was attributed to Cu_2_O. Scherrer analysis of the Cu(111) peak showed a crystallite size of 30.8 nm. Similar to the PtNTs, there were no remaining Fe peaks, which suggests the near complete replacement of Fe with Cu and is consistent with the EDS and ICP-OES. As was the case with the FeNWs, the PtNTs and CuNWs have feature sizes seen in SEM images that are larger than the observed XRD crystallite sizes. These findings show that the various aerogels are polycrystalline in nature, which can have implications for their reactivity and electrochemical properties.

The crystallinity of the various aerogels was further assessed using TEM and SAED, as shown in Figure 4c–h. The TEM images reveal regions of both amorphous and crystalline materials, thus confirming the polycrystallinity as observed by XRD. Moreover, the observed length scales of the various crystalline domains are commensurate with crystallite sizes predicted through Scherrer calculations. The SAED lattice spacings observed (Table S7) for the FeNWs matched those expected for cubic Fe. For the CuNWs, the lattice spacings indicated mixed cubic and hexagonal Cu but no presence of CuO or Cu_2_O. For the PtNWs, the SAED suggests the presence of multiple crystal phases that could correlate with cubic Pt and FePt alloys (FePt and FePt_3_). Both EDS and ICP-OES confirmed that some residual Fe was present in the PtNTs, which could account for the observed FePt and FePt_3_ lattice spacings. Finally, the increased electron density along the longitudinal axis edges of the CuNWs and PtNTs further suggests the hollow core and outward growth mechanism we propose for the galvanic displacement reaction.

**Figure 5:**
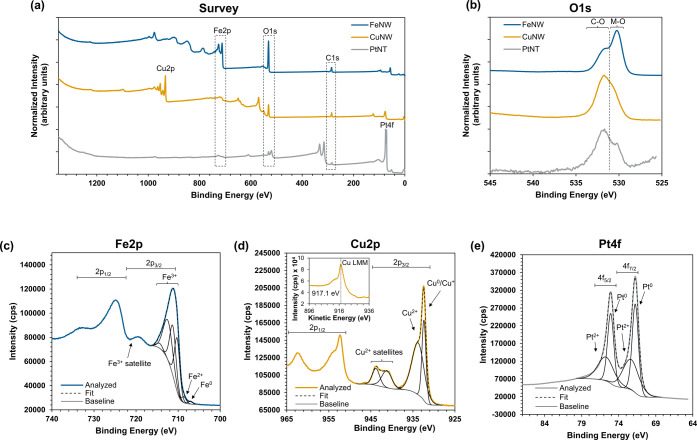
XPS data of the FeNWs produced through magnetic field-assisted synthesis in a 150 mT field and galvanic displacement. Survey scans (a) and high resolution O 1s scans (b) of the FeNWs, CuNWs and PtNTs, a high resolution Fe 2p scan of the FeNWs (c), a high resolution Cu 2p scan (d) and Cu LMM Auger scan (inset) of the CuNWs, and a Pt 4f high resolution scan of the PtNTs (e).

Figure 5 presents the XPS spectra of the FeNWs, CuNWs, and PtNTs. For all samples, adventitious carbon was used as an internal standard to adjust the binding energy. Tables reporting full analysis and fit details of the survey and high-resolution scans are reported in the Supporting Information (Tables S8–S11). The survey scans indicate the presence of metal species, oxygen, and adventitious carbons across all three samples. The trace amount of Cl detected in the CuNWs and PtNTs is attributed to residual precursor from galvanic displacement or the chemical reduction of FeCl_3_. For the FeNWs, the characteristic Fe 2p peak indicated the presence of Fe at the surface. Following galvanic displacement with Cu or Pt, this Fe 2p peak is suppressed, and the Cu 2p peak or Pt 4f peak appears, respectively. This indicates the replacement of Fe with the displacing metals and supports the results previously described for EDS, ICP-OES, and XRD. Oxygen was observed in the survey scans of all three samples. The O 1s high resolution scans of the samples (Figure 5b) show spectral features both in the metal–oxygen (529–530 eV) and carbon–oxygen (531–532 eV) bond regions.^24,56^ This suggests that some of the observed oxygen content originates from oxygen-containing surface species like adventitious carbon but also indicates the presence of some surface oxidation of the metals.

The surface oxidation is further supported by high-resolution scans for the various metals. The deconvoluted Fe 2p high-resolution scan of the FeNWs shows peaks indicative of both metallic Fe (707.1 eV) and Fe^2+^ (708.7 eV).^56^ Multiplet splitting and satellite peaks characteristic of Fe^3+^ were also observed.^57^ The Cu 2p scan shows that the CuNWs have surface oxides. The peak at 932.4 eV can indicate the presence of both metallic and cuprous Cu. The peak observed at 933.8 eV and the presence of Cu^2+^ shake-up peaks at 941.2 and 943.8 eV are indicative of the presence of cupric species on the surface of the CuNWs. The Cu LMM Auger scan (Figure 5d; inset) also supports the observation of valent copper and has a profile shape similar to a Cu^2+^ and Cu^+^ species with a peak kinetic energy of 917.1 eV.^56,58^ The Pt 4f high-resolution scan shows that the PtNTs have both Pt^0^ (71.8 eV) and Pt^2+^ (72.3 eV) observed at the surface, with metallic Pt accounting for a slightly higher fraction of the overall Pt. XPS is a surface-sensitive technique, while XRD measures the bulk samples, so it is not surprising that oxides were observed by XPS. The lack of metal oxide observed by XRD for the FeNWs and PtNTs implies that the metal oxide is only present on the surface and is not part of the bulk material. For the CuNWs, the Cu_2_O observed by XRD can be further oxidized to CuO at the surface,^59^ which could account for the Cu^2+^ observed by XPS. Additionally, the polycrystallinity of the materials could contribute to their susceptibility to surface oxidation.^59,60^

**Figure 6:**
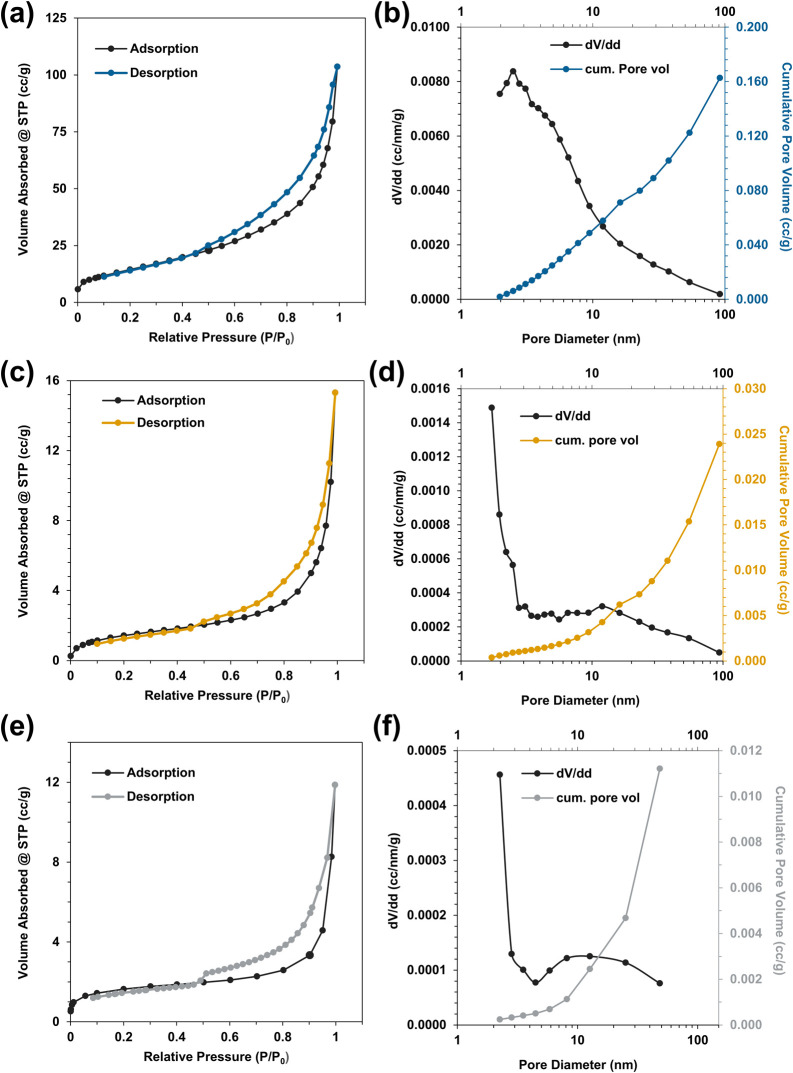
Nitrogen gas adsorption–desorption isotherms, cumulative pore volumes, and pore size distributions for FeNW (a, b), CuNW (c, d), and PtNT (e, f).

Nitrogen adsorption–desorption curves were collected for all three samples, and Brunauer–Emmett–Teller (BET)^61^ analysis was done to analyze the sample surface areas while Barrett–Joyner–Halenda (BJH)^62^ analysis was used on the adsorption curves to determine the pore volumes and pore diameters, as shown in Figure 6. All three isotherms have similar shapes to the IUPAC type IV isotherms, which is indicative of a mesoporous structure and features hysteresis due to pore condensation within the mesopores.^63,64^ The curves all have an IUPAC type H3 hysteresis shape,^63^ which has no plateau-like behavior upon desorption and indicates an immediate decrease in the amount of adsorbed nitrogen as the pressure is decreased. This type of shape can be observed if there are macropores present that are not completely filled with pore condensate.^63^ This is consistent with the SEM micrographs that show macroporous regions of empty spaces between the intertangled nanowires. The BET specific surface area for FeNWs is 54.1 m^2^/g, correlating to an idealized geometry of an approximately 14 nm sphere. Following galvanic displacement to form CuNWs and PtNTs, the surface area decreases significantly. The CuNWs have a surface area of 5.4 m^2^/g, which correlates to an idealized geometry of an approximately 125 nm sphere, while the PtNTs have a surface area of 5.8 m^2^/g, which correlates to an idealized geometry of a 48 nm sphere. Despite the relatively complex structure of the nanowires and nanotubes within the aerogels compared to an idealized sphere, the spherical diameters calculated from the BET measurements follow the same trend in feature sizes measured from the image analysis of the SEM micrographs. The FeNWs had the smallest diameters, which increased following galvanic displacement with both copper and platinum. The PtNTs had an intermediate-sized diameter, and the CuNWs had the largest. BJH analysis of the three samples shows that the galvanically displaced samples had lower pore volumes and larger pore diameters compared to those of the FeNW aerogels. The decrease in CuNWs' specific surface area compared to FeNWs is attributed to the larger outer diameter of 228 nm compared to the average FeNW diameter of 95 nm (Figure 3), along with the smoother CuNW surface area compared to the rough beads-on-a-string structure of FeNWs. The decrease of the PtNT surface area is attributed to the nearly three times higher bulk metal density of Pt compared to Fe (21.45 g/cm^3^ compared to 7.87 g/cm^3^).

**Figure 7:**
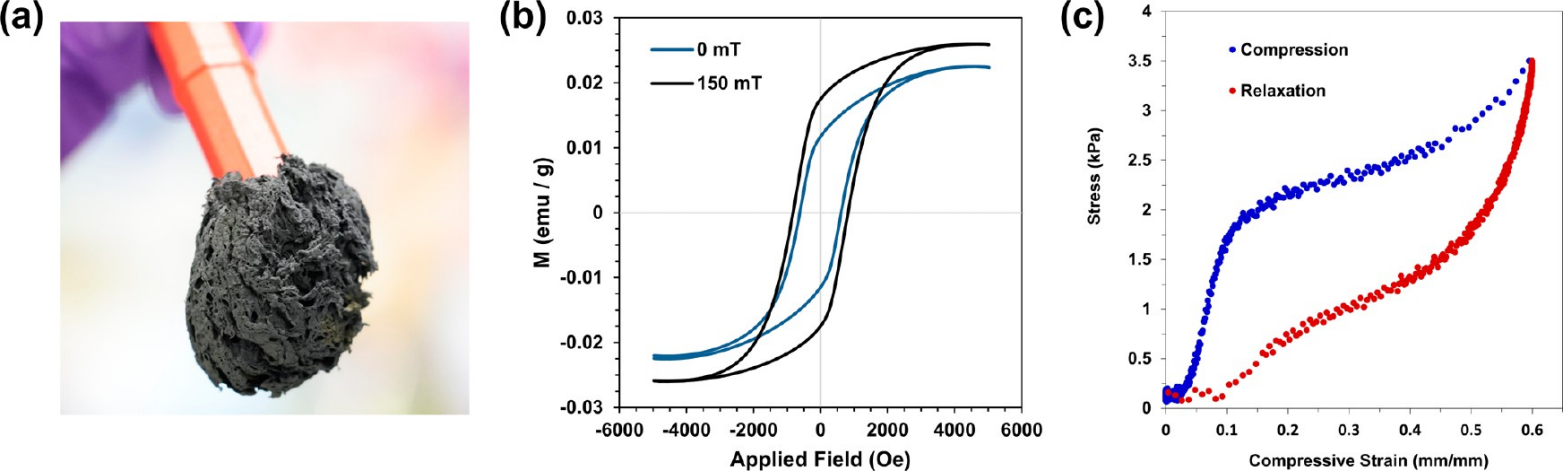
Photograph of an FeNW supercritical dried aerogel attached to a stir bar magnet (a). Vibration sample magnetometry (VSM) hysteresis curves for FeNW aerogels prepared in the presence of 0 and 150 mT fields (b). Representative stress–strain curve of an FeNW aerogel (c).

Figure 7 shows magnetic and mechanical response data that further support the concept of applying the FeNW aerogels as a multifunctional material. FeNW aerogels were attracted to small magnets, as seen in Figure 7a. Their magnetic properties were measured through vibrating sample magnetometry (VSM), and the magnetic hysteresis curves for the zero field and 150 mT samples are shown in Figure 7b. The VSM data for the samples prepared at lower applied magnetic field strengths are shown in Figure S10. For all samples, the measurements were collected in triplicate, and the reported plots are the average of the three samples. All samples show characteristic hysteresis loops of a ferromagnetic material. For all samples, the specific saturation (*M*_s_) and remnant (*M*_r_) magnetizations were similar, with some variability from measurement to measurement. This is best understood by considering that the FeNWs consist of localized bundles of ordered nanowires but on a global scale have some degree of randomization, as previously discussed in Figure 2. This randomization can cause lower saturation magnetizations to be observed due to magnetic domains in opposite directions canceling each other out.^43^ Additionally, since *M*_s_ is dependent on the particle size and crystallinity,^55^ it is not surprising that the samples will have similar magnetic properties due to their similar diameters and crystallite sizes, as shown in Figures 3a and 4a, respectively. The coercivities, however, did indicate some dependence on applied field strength during synthesis, with the 150 mT FeNWs showing a much higher coercivity than all other samples, similar to other reports of magnetic iron nanowires.^65^

After supercritical drying, iron nanowire aerogels were observed to have a similar mechanical behavior as a cotton ball, demonstrating the ability to compress and relax back to their original shape (see Video S2). To estimate the elastic modulus, a compressive stress–strain curve was initially collected with a 500 N load cell universal testing instrument, but the response was less than the instrument's sensitivity range. The representative stress–strain curve in Figure 7c was collected using a parallel plate rheometer, which indicated a yield strength of approximately 1.7 kPa before plastic deformation at higher strains. The estimated elastic modulus from the linear portion of the curve at strains up to 0.1 was 23.4 kPa in the range of many organosilicon-based aerogels.^66^ The toughness determined by the area under the curve up to a compressive strain of 0.6 was 1.3 kJ/m^3^, with 0.65 kJ/m^3^ retained upon relaxation back to 0 strain. The compressibility and relaxation of the iron aerogels support the ability to conform them into arbitrarily shaped films seen in Figure 1, thus overcoming a significant challenge of brittle metal aerogels.

**Figure 8:**
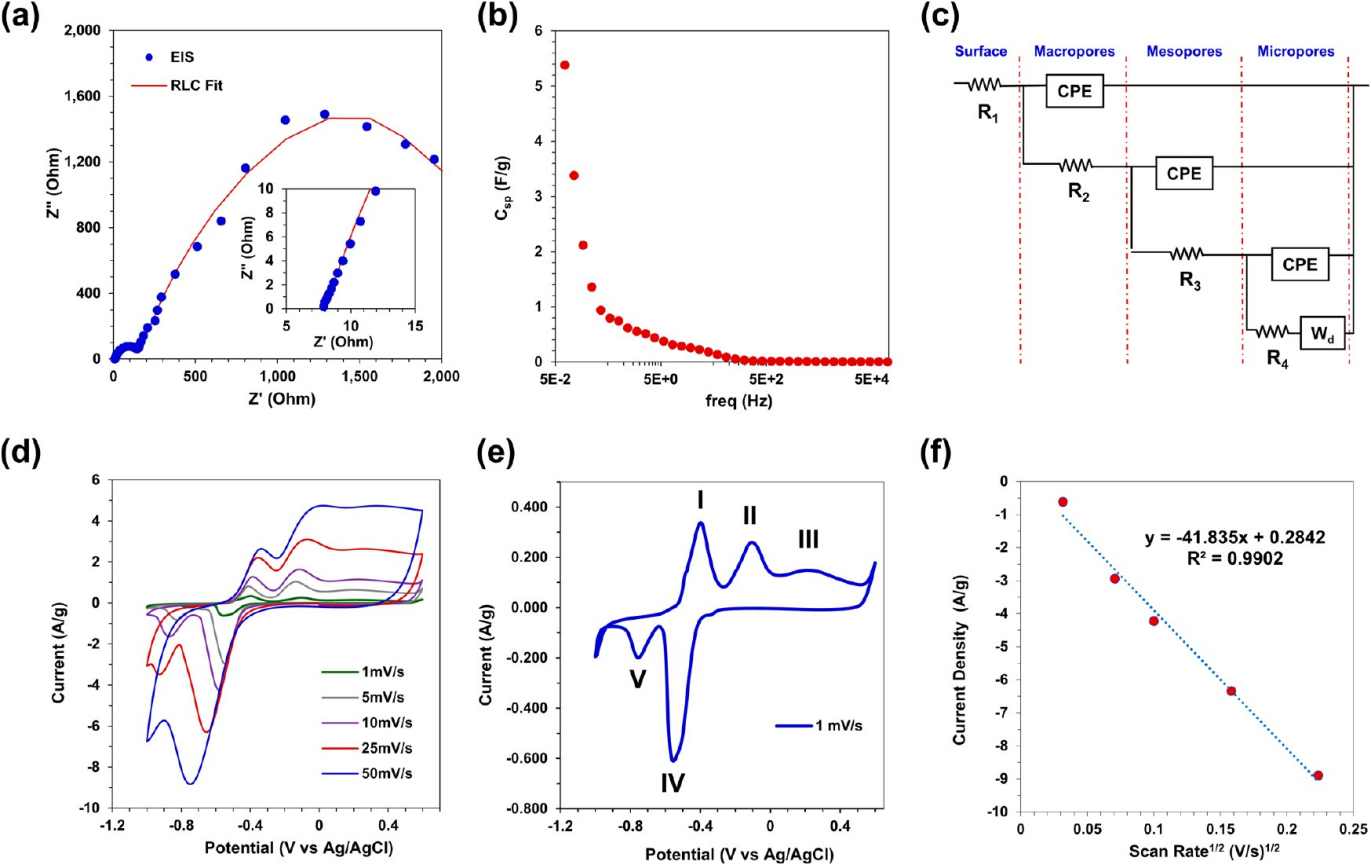
Electrochemical characterization of CuNWs was performed in 1 M KOH for electrochemical impedance spectroscopy (a–c) and cyclic voltammetry (d–f). EIS spectra (blue data points) are shown with the high frequency range in the inset; RLC fit is overlaid in red. The specific capacitance, *C*_sp_, for each impedance data point from (a) is shown in (b) with the EIS fit model (c). Cyclic voltammetry curves for scan rates of 1, 5, 10, 25, and 50 mV/s (d). CV curve for 1 mV/s is shown separately (e). Current density versus (scan rate)^1/2^ for the reduction peaks at approximately −0.5 V (f).

To assess the potential of CuNW films as free-standing, binderless, 3-dimensional capacitive electrodes, galvanostatic electrochemical impedance spectroscopy and cyclic voltammetry were performed in 1 M KOH electrolyte. The EIS spectrum with a high frequency inset shown in Figure 8a reveals a steep capacitive rise at high frequencies, then the formation of a depressed semicircle at midfrequencies before exhibiting further capacitance and diffusion resistance at lower frequencies. A second semicircle appears at low frequencies with a peak at approximately 0.2 Hz. This frequency response behavior is characteristic of porous electrodes with a transmissive boundary and is fitted with a transmission line model^67,68^ shown in Figure 8c with parallel resistors and constant phase elements for multiple levels of electrode pore structure (see Supporting Information for detailed fitting information). The impedance data in Figure 8a was used to calculate the specific capacitance, *C*_sp_, at each frequency using the relationship, *C*_sp_ = 1/(2π*fZ*”*m*), where *f* is the scan frequency, *Z*” is the imaginary impedance, and *m* is sample mass; this is plotted in Figure 8b. *C*_sp_ based on impedance data at 0.07 Hz is 5.4 m^2^/g, which closely matches the specific surface area estimate of 5.4 m^2^/g based on nitrogen gas adsorption–desorption in Figure 6. Cyclic voltammetry curves for CuNWs across a range of scan rates from 1, 5, 10, 25, and 50 mV/s are shown in Figure 8d. The 1 mV/s scan is shown separately in Figure 8e with anodic and cathodic redox peaks exhibiting hysteresis with increasing scan rates expected for porous materials. Anode peaks reflect the (I) Cu/Cu^+^, (II) Cu/Cu(OH)_2_, and (III) Cu(OH)_2_/Cu redox couples, while cathodic peaks correspond to the (IV) Cu_2_O/Cu and (V) Cu(OH)_2_/Cu couples.^69^ The current density versus scan rate^1/2^ for the reduction peak at approximately −0.5 V (vs Ag/AgCl) in Figure 8f demonstrates the reversibility of the surface redox activity. EIS and CV data indicate that the CuNW films are conductive and capacitive and offer good solvent accessibility.

**Figure 9:**
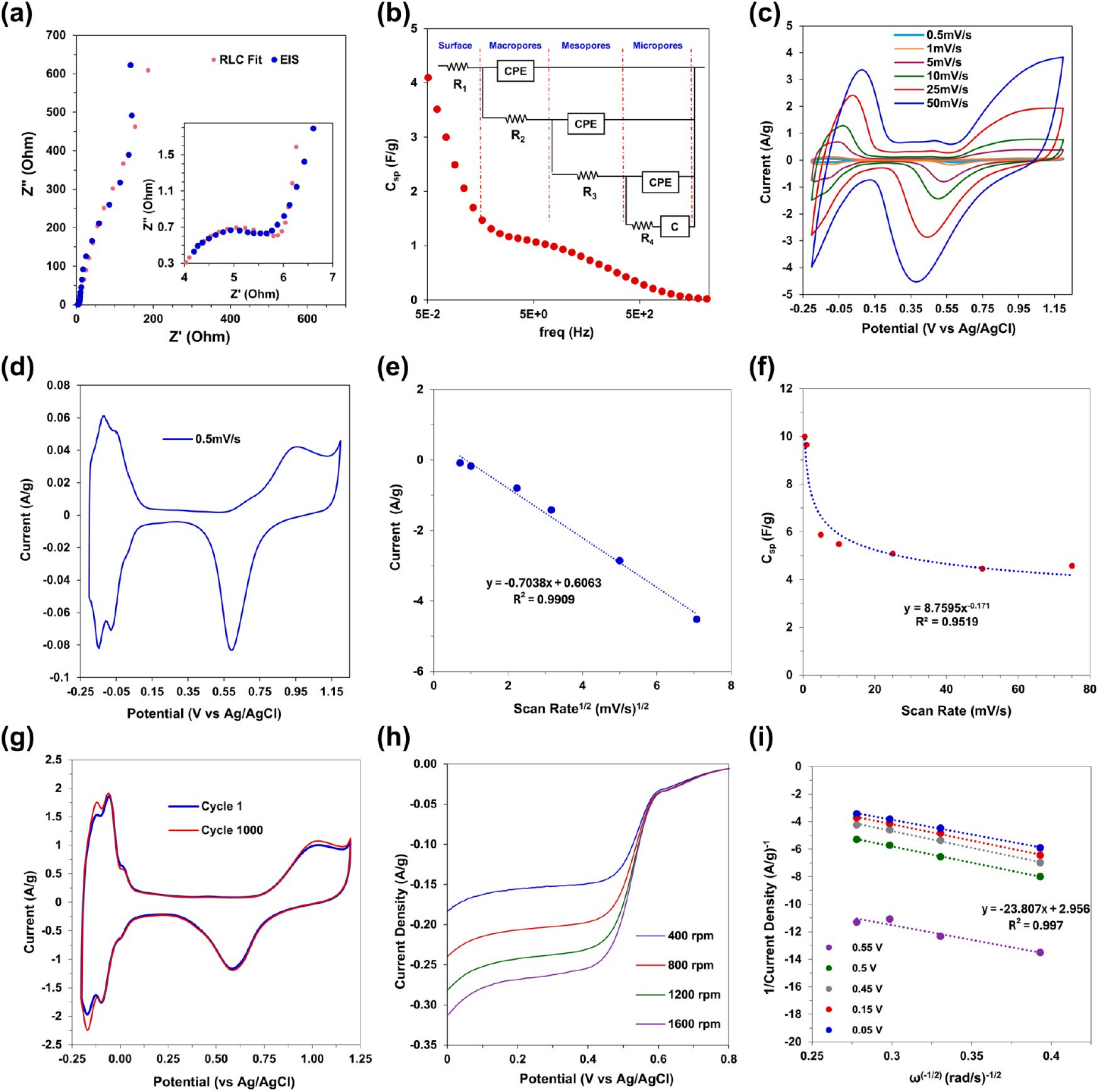
Electrochemical characterization of PtNTs was performed in 0.5 M H_2_SO_4_ for electrochemical impedance spectroscopy (a, b) and cyclic voltammetry (c–g) and rotating disk electrode linear sweep voltammograms (h, i). EIS spectra in blue data points is shown with the high frequency range in the inset; RLC fit is overlaid in red (a). The specific capacitance, *C*_sp_, for each data point is shown in (b) with the RLC fit model in the inset. Cyclic voltammetry curves for scan rates of 0.5, 1, 5, 10, 25, and 50 mV/s (c). CV curve for 0.5 mV/s is shown separately (d). Current density versus (scan rate)^1/2^ for the Pt reduction peaks at approximately 0.4–0.5 V (vs Ag/AgCl) (e). Specific capacity, *C*_sp_, determined from the nonfaradaic region in (c) at approximately 0.2 V (vs Ag/AgCl) (f). The 1st and 1000th cycles of cyclic voltammetry were performed at 25 mV/s (g). Linear sweep voltammetry curves from 0.8 to 0 V (vs Ag/AgCl) conducted in oxygen saturated electrolyte (h). The reciprocal of current density from (h) versus (scan rate)^−1/2^ (i).

To determine the catalytic potential of PtNT free-standing films, galvanostatic electrochemical impedance spectroscopy (GEIS) and cyclic voltammetry were performed in 0.5 M H_2_SO_4_ electrolyte to assess the material and mass transfer impedances as well as the electrochemically active surface area (ECSA). As seen in the EIS spectrum in Figure 9a, a depressed semicircle at high frequencies transitions to mass transfer impedance at intermediate frequencies and then quickly rises with an increase in capacitance at lower scan frequencies. Like the CuNW EIS spectrum, *C*_sp_ values were calculated for PtNTs and plotted versus the scan frequency (Figure 9b). The specific capacitance determined at 0.05 Hz is 4.1 F/g, equivalent to an approximately 28 nm idealized spherical nanoparticle. The Pt EIS spectrum was fitted with a transmission line model (TLM) of parallel resistors and constant phase elements for each level of porosity (Figure 9b inset) to include between nanotubes (seen in Figure 3f), the open channel inside the nanotubes where the iron nanowire template dissolved out (seen in Figure 3g), and between nanoparticles within the cylindrical platinum shell. See the Supporting Information for fitting details and model constants.

Cyclic voltammetry of free-standing PtNT films was performed for scan rates of 0.5, 1, 5, 10, 25, and 50 mV/s shown in Figure 9c, with the 0.5 mV/s scan shown separately in Figure 9d. The CV scans reveal the expected oxidative toe region above 0.5 V (vs Ag/AgCl) and the corresponding platinum reduction peak, along with hydrogen adsorption and desorption peaks below 0 V. The plot of the current density of the Pt reduction peak versus scan rate^1/2^ shown in Figure 9e indicates good surface redox reversibility. The *C*_sp_ determined from the nonfaradaic region at approximately 0.2 V (vs Ag/AgCl) for the CV scans in Figure 9c is shown in Figure 9f. The *C*_sp_ for the 0.5 mV/s CV scan is 9.99 F/g. The electrochemically accessible surface area (ECSA) determined from the hydrogen desorption peaks of the 0.5 mV/s CV scan indicated a capacitance of 2.78 F/g. Given a metal surface capacitance of 30 μF/cm^2^,^70^ the ECSA of PtNTs is 13.9 m^2^/g, or slightly more than double the specific surface area indicated by BET. This increased surface area may likely be due to the dissolution of any remaining Fe in the presence of the acidic electrolyte, allowing access to additional pores within the nanotube sidewalls. The ECSA is similar to that achieved by free-standing platinum macrotube films chemically reduced from insoluble salt needles,^71,72^ and close to the specific surface area of commercial platinum black at approximately 25 m^2^/g. Further increases of platinum specific surface area and capacitance might be achieved through a partial galvanic displacement similar to the approach by Alia and colleagues, who displaced commercial nickel nanowires with platinum.^73^

Surface stability of the PtNTs was tested by conducting 1000 CV cycles at 25 mV/s, with the first and 1000th cycle superimposed on Figure 9g with little change in peak positions or magnitudes. Linear sweep voltammetry seen in Figure 9h was used to assess the PtNT films for oxygen reduction reaction (ORR) electrode applications by using 0.5 M H_2_SO_4_ electrolyte solutions with saturated oxygen. Pressed PtNT films were adhered to glassy carbon electrodes (GCE) using stock 5% Nafion solutions and ambiently dried (EIS and CV curves shown in Figure S11a,b; 5% Nafion only on a GCE was also tested to determine background capacitance, shown in Figure S11c). LSV curves were initially collected with a 10 mV/s sweep rate, but minimal ORR current density was observed (Figure S11d). A LSV sweep rate of 0.5 mV/s was used to facilitate any reactant from the film surface exposed to bulk electrolyte into the film pore network, and LSV scans are shown in Figure 9h. LSV curves swept from 1.2 to 0 V (vs Ag/AgCl), and current density is seen to significantly increase at approximately 0.6 V and then plateau between 0.4 to 0.1 V (vs Ag/AgCl). At voltages below 0.1 V current density magnitude rises again as hydrogen adsorbs on the electrode surface. The Koutecky–Levich plot in Figure 9i shows the reciprocal of the current density plotted versus rotation speed to the negative one-half power, ω^–1/2^. The slope of the linear regression of the data points at 0.15 V indicates a 3.28 electron process. This suggests the PtNT films are able to mediate close to the preferred ORR half reaction of O_2_ + 4H^+^ + 4e^–^ → 2H_2_O.

## Conclusions

In conclusion, we have demonstrated magnetic-field-assisted (MFA) synthesis to prepare FeNW gels and aerogels that can be conformed into arbitrary shapes and thin films. Prior to FeNW gel shape formation, FeNWs could serve as a sacrificial template for galvanic displacement and allowed the formation of CuNW and PtNT gels and aerogels. We showed that the applied magnetic field strength influenced the alignment of the FeNWs, with higher field strengths resulting in a higher orientation order parameter, and zero field conditions resulting in low nanowire ordering. These FeNWs had ferromagnetic properties with higher coercivities observed in samples prepared with the highest applied magnetic fields. Mechanical compression and relaxation of the FeNW aerogels enable conformation of them into arbitrary geometries and suggest a ductility not commonly seen in metal aerogels. The FeNWs had consistent nanowire diameters, crystallite sizes, and saturation and remnant magnetizations regardless of the applied magnetic field strength, which supports the proposed beads-on-a-string mechanism of initial formation of small iron nanoparticle clusters that are aligned by the externally applied magnetic field and coalescence into locally aligned nanowires upon subsequent chemical reduction of additional iron ions. Galvanic displacement to CuNWs and PtNTs allowed nearly complete replacement of the iron with the displacing metals, as shown by EDS and ICP-OES measurements. SEM micrographs and size analysis of the displaced nanostructures suggest an outward growth mechanism with hollow inner shells. Electrochemical studies allowed for assessment of the CuNWs and PtNTs as potential 3D electrodes for capacitive energy storage or electrocatalytic applications. In general, the MFA synthesis and subsequent galvanic displacement allowed for a rapid and template-free synthesis of metal aerogels that were conformable to various shapes or free-standing films without the need for any additional binding material. This method is envisioned as an avenue to prepare many other types of 3D metal electrodes for a broad range of energy storage, catalysis, and sensing applications.

## Supporting Information Available

Fe aerogel chemical reduction (MP4)
Fe aerogel mechanical compression (MP4)
Experimental synthesis, image analysis, inductively coupled plasma optical emission spectroscopy data, SEM images of CuNWs, PtNTs and galvanic displacement to gold nanowires, vibrating sample magnetometry data, EIS fitting, and XPS fitting (PDF)

## Abbreviations

FeNW: iron nanowire
CuNW: copper nanowire
PtNT: platinum nanotube
3D: three-dimensional
MEMS: microelectromechanical systems
AAO: anodic aluminum oxide
MFA: magnetic-field-assisted
SEM: scanning electron microscopy
EDS: energy dispersive X-ray spectroscopy
OOP: orientation order parameter
XRD: X-ray diffractometry
XPS: X-ray photoelectron spectroscopy
VSM: vibrating sample magnetometry
ICP-OES: inductively coupled plasma optical emission spectroscopy
RLC: resistor-inductor-capacitor
NW: nanowire
NT: nanotube
GEIS: galvanostatic electrochemical impedance spectroscopy
CV: cyclic voltammetry
ESCA: electrochemically active surface area
TLM: transmission line model
