# Supplementary material for: Magnetic-Field-Assisted Fe Nanowire Conformable Aerogels Galvanically Displaced to Cu and Pt for Three-Dimensional Electrode Applications
Source: ACS Appl Mater Interfaces. 2025 Apr 28;17(18):26854–70. doi: 10.1021/acsami.5c00693 (PMC12067370; doi:10.1021/acsami.5c00693)
Supplement: Supplementary file 3 — am5c00693_si_003.pdf [file am5c00693_si_003.pdf]

Magnetic-Field-Assisted Fe Nanowire Conformable Aerogels Galvanically Displaced to Cu and Pt for 3-Dimensional Electrode Applications

Rosemary L. Calabro [1,2], Garret L. Longstaff [1], Edward M. Tang [1], Veronika M. Xiao [1], Alexa S. Zammit [1], Felita W. Zhang [1], Enoch A. Nagelli [1,3], Peter H. Chapman [4], Timothy J. Lawton [5], Mark A. Allen [6], Anchor R. Losch [1], Jesse L. Palmer [1], Alexander D. Ciampa [1], Ian Z. Burpeau [1], Veronica M. Lucian [1], Galen T. Mandes [1], Stephen F. Bartolucci [2], Joshua A. Maurer[2], F. John Burpo[1,3,]*

[1] Department of Chemistry and Life Science, United States Military Academy, West Point, NY 10996 USA
[2] U.S. Army Combat Capabilities Development Command-Armaments Center, Watervliet Arsenal, NY 12189, USA
[3] Photonics Research Center, United States Military Academy, West Point, NY 10996 USA
[4] Department of Physics and Nuclear Engineering, United States Military Academy, West Point, NY 10996 USA
[5] U.S. Army Combat Capabilities Development Command-Soldier Center, Natick, MA 01760, USA
[6] U.S. Army Combat Capabilities Development Command-Army Research Laboratory, Aberdeen Proving Ground, MD 21005

Corresponding Author

F. John Burpo – Department of Chemistry and Life Science, United States Military Academy, West Point, NY 10996 USA; Photonics Research Center, United States Military Academy, West Point, NY 10996 USA; ORCID 0000-0002-0303-1043; Email: john.burpo@westpoint.edu

Synthesis Details

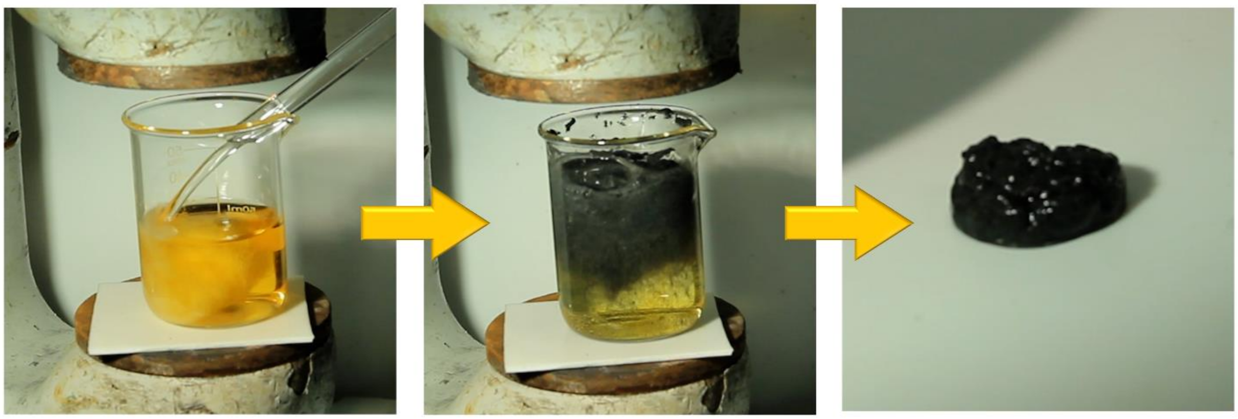

Figure S1. Synthesis of iron nanowires prepared in a 150 mT magnetic field. The synthesis is done by adding 25 mL of 100 mM $NaBH_4$ via a funnel to 30 mL of 100 mM $FeCl_3{\cdot}6H_2O$. Chemical reduction requires 1-2 minutes before the iron nanowire gel may be transferred for rinsing with deionized water and solvent exchanged with ethanol or drying at ambient temperature. Gels may be pressed into any arbitrary film thickness and shape.

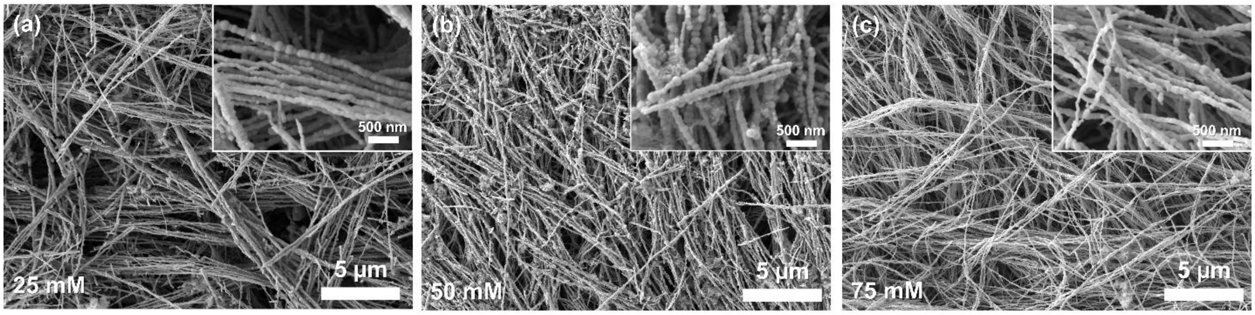

Figure S2. FeNWs synthesized with 25 mM (a), 50 mM (b), and 75 mM (c) $FeCl_3{\cdot}6H_2O$ and chemically reduced with 100 mM $NaBH_4$.

Vacuum-assisted flow-through galvanic displacement apparatus

Galvanic displacement allowed exchange of iron with metals that have higher reduction potentials such as copper or platinum. First a FeNW aerogel was synthesized in a magnetic field. In a typical procedure 25 mL of 100 mM $FeCl_3 \cdot 6H_2O$ was placed in a 150 mT magnetic field. Rapid reduction of the Fe^{3+} ions occurred upon addition of 30 mL of 100 mM $NaBH_4$. The reaction was allowed to continue for 2 minutes resulting in a black gel consisting of a porous agglomerate of magnetically aligned FeNWs. The gel was then transferred to another container and was soaked in 100 mM $NaBH_4$ to reduce any remaining Fe^{3+} ions. The gel was then transferred to a filter funnel and was rinsed with at least 100 mL of deionized water to remove any remaining salt or reducing agent solution.

The FeNW gel could then serve as a shape scaffold for galvanic displacement. The FeNW gel was transferred to a 5 mL syringe that was fit with a steel mesh at the bottom (Figure S3). The syringe was carefully tapped until the FeNW gel was sealed around the walls of the syringe and then the syringe was inserted into a vacuum flask with a rubber septum. Then, gentle vacuum was applied and 10 mL of either 500 mM $CuSO_4$ or 100 mM K_2PtCl_4 was added to the syringe. Flowing the oxidizing agent through a nanowire array using vacuum pressure allowed increased smoothness and uniformity across the gel structure compared to other methods of galvanic displacement, such as passive diffusion. Finally, the galvanically displaced gel was rinsed with about 50 mL of deionized water. Samples stored for later analysis were then rinsed with about 25 mL of deaerated ethanol to prevent oxidation. The gel could then be removed and either pressed into a thin film, or supercritical dried into an aerogel.

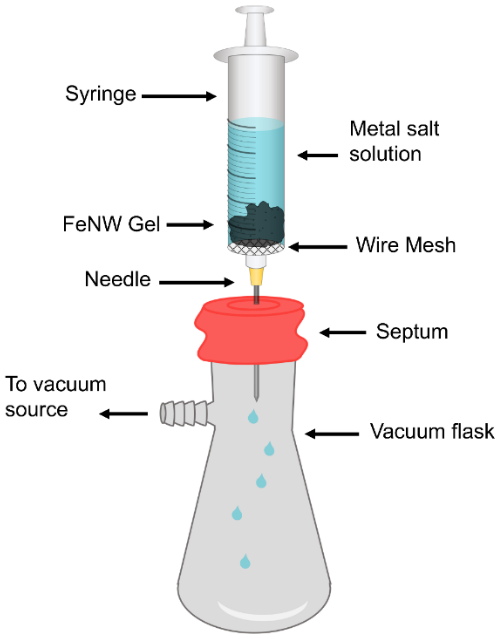

Figure S3. Schematic representation of the galvanic displacement apparatus used to displace iron nanowires with copper or platinum.

ImageJ analysis

The freely available ImageJ software package[1] was used to determine the feature sizes of iron nanowire diameters, lengths, and nanotube cross-sectional thickness distributions.

Nanowire Orientation Color-Map Generation

Color-mapped images based on orientation angle were generated with the OrientationJ plugin (Biomedical Image Group, EPFL, Switzerland) for ImageJ. Individual nanowires are assigned a hue dependent on their orientation through a sliding Gaussian analysis window.[2]

Nanowire Orientation Analysis:

Orientation Order Parameter (OOP) was quantified from SEM images to compare nanowire alignment between magnetic field strengths. GTFiber was used to calculate OOP for nanowire arrays through a five-step preprocessing workflow that includes diffusion filtering, top hat filtering, thresholding and cleaning, skeletonization, and orientation mapping.[3] Following the preprocessing, local OOP values are calculated across the images in square subsections of incrementing frame sizes. Initial hyperparameters of 2500 nm grid step, 1250 nm frame step, 20 nm Gaussian smoothing, 100 nm orientation smoothing, 5 s diffusion time, 30 nm top hat size, 0.3 global threshold, 500 nm^2 noise max area, and 100 nm fringe removal were selected for images with a zoom factor of 5000x. These hyperparameters were subsequently scaled to maintain the ratios for images with different magnifications. Outputs were OOP values as a function of frame size (S_{2D}) modeled by an exponential decay function, the length scale of OOP decay (λ_C), and the asymptotic OOP (S_{Full}). For each field strength, images with asymptotic OOP values outside of two standard deviations of the mean were considered outliers and removed from analysis. OOP values as a function of synthesis magnetic field strength and OOP analysis frame size are summarized in Table S1 below.

Table S1. Orientation order parameter (OOP) average values and standard deviations across the range of synthesis magnetic field strengths and analysis frame sizes.

	Analysis Frame Size (µm)					
**Field (mT)**	**0.5**	**1**	**1.5**	**1.75**	**2**	**Full**
**0**	0.67±0.038	0.51±0.119	0.42±0.145	0.39±0.146	0.36±0.145	0.14±0.142
**9**	0.64±0.049	0.53±0.107	0.46±0.091	0.43±0.079	0.41±0.075	0.28±0.071
**19**	0.85±0.038	0.73±0.170	0.63±0.153	0.60±0.132	0.56±0.124	0.20±0.117
**37**	0.86±0.016	0.75±0.022	0.66±0.034	0.63±0.039	0.60±0.040	0.24±0.041
**75**	0.93±0.028	0.88±0.013	0.84±0.013	0.82±0.016	0.80±0.016	0.61±0.018
**150**	0.94±0.072	0.89±0.113	0.84±0.133	0.82±0.131	0.80±0.128	0.56±0.124

Table S2. Orientation order parameter (OOP) average values and standard deviations across the range of synthesis magnetic field strengths and analysis frame sizes. Nanowires and nanowire clusters with a diameter greater than 150 nm were excluded.

	Magnetic Field Strength						
**Diameter (nm)**	**0 mT**	**9 mT**	**19 mT**	**37 mT**	**74 mT**	**150 mT**	**Global**
**Mean**	71.1	82.6	83.3	81.1	73.2	95.4	79.0
**Std Dev**	18.2	22.1	16.7	20.2	17.1	23.8	20.9

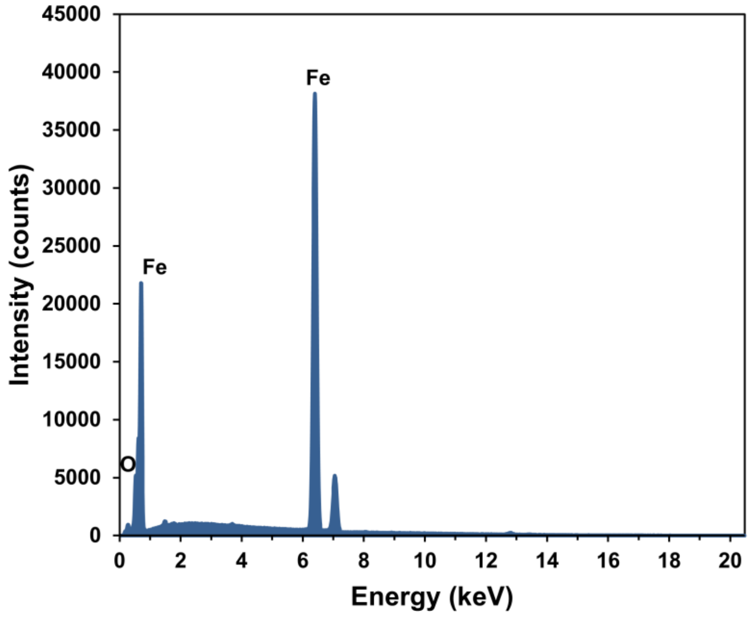

Figure S4. Energy dispersive X-ray spectroscopy (EDS) of an FeNW aerogel.

Inductively coupled plasma optical emission spectroscopy (ICP-OES) measurements

Elemental composition of the FeNWs, CuNWs, and PtNTs was assessed via ICP-OES measurements. Three of each type of sample was dissolved in HNO_3 and then diluted to 7 mg/L in 5% HNO_3. Each sample was measured in triplicate and compared to calibration standards. Fe was measured at 238.204 nm, Cu at 327.393 nm, and Pt at 265.945 nm. Linear regressions were run on the calibration standards and used to determine the concentration and weight fraction of each metal.

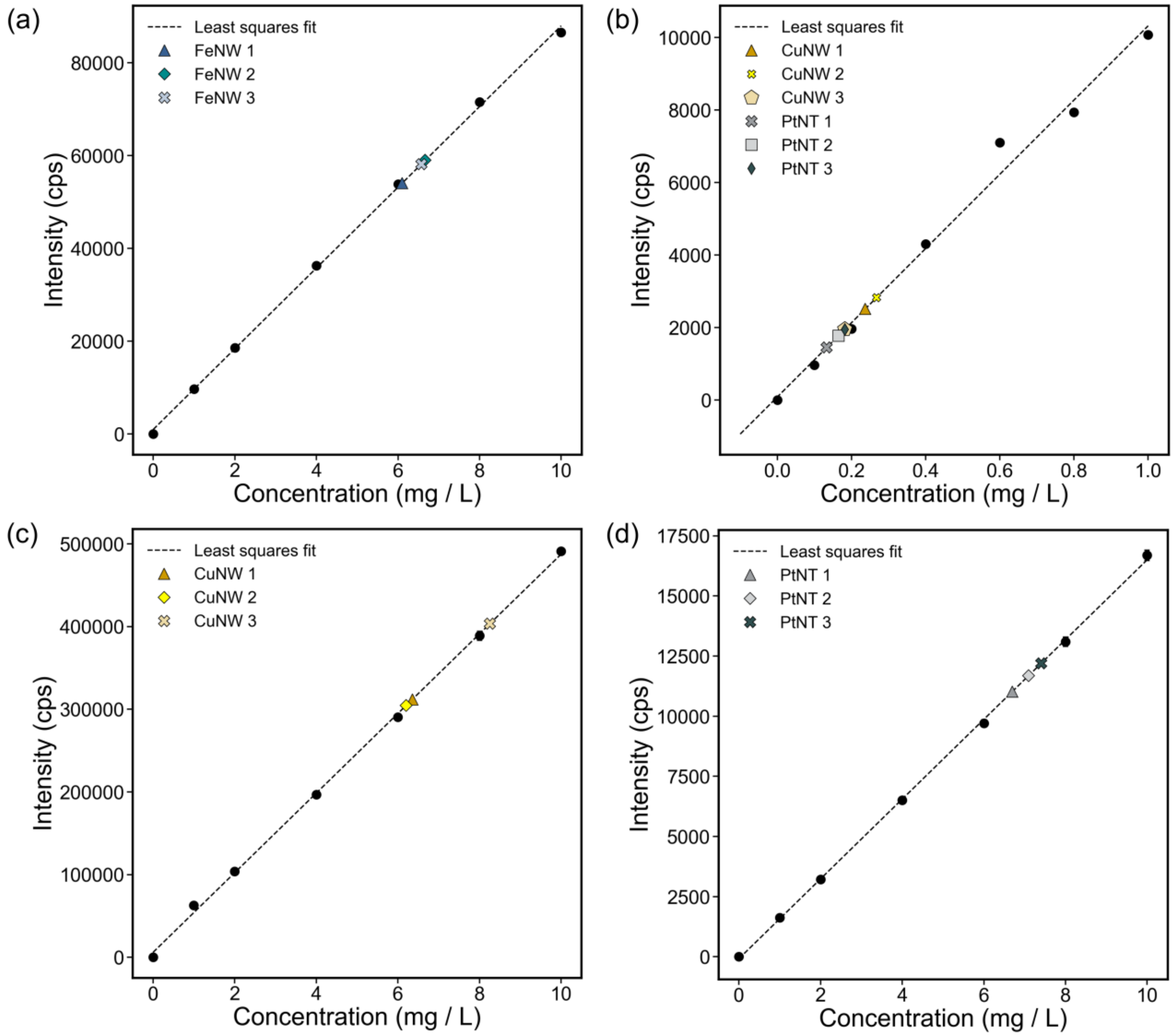

Figure S5. Inductively coupled plasma optical emission spectroscopy for the various samples. Fe concentration for the FeNWs measured at 238.204 nm (a), Fe concentration for the CuNWs and PtNTs measured at 238.204 nm (b), Cu concentration for the CuNWs measured at 327.393 nm (c), and Pt concentration for the PtNTs measured at 265.945 nm (d). The black circles represent measurements for the calibration standards.

Table S3. Linear regression fit parameters of the Fe, Cu, and Pt calibration curves for the ICP-OES measurements. The measurements were fit to the linear equation y = mx + b where m represents the slope and b the y-intercept.

Analyte	m	b
**Fe 238.204 nm (0-10 mg/L)**	8699.327 ± 107.134	972.2227 ± 601.9694
**Fe 238.204 nm (0-1 mg/L)**	10232.67 ± 490.437	87.54812 ± 275.569
**Cu 327.393 (0-10 mg/L)**	48144.43 ± 610.4222	5876.135 ± 3429.868
**Pt 265.945 (0-10 mg/L)**	1658.286 ± 13.95891	-82.8809 ± 78.43298

Table S4.. Measured ICP-OES intensities and calculated weight fractions for Fe, Cu and Pt in the FeNW aerogels and galvanically displaced samples.

Sample	FeNW	CuNW		PtNT	
**Analyte**	Fe	Fe	Cu	Fe	Pt
**Average Intensity (CPS)**	57053 ± 2626	2669 ± 220	308113 ± 5295	1614 ± 229	11350 ± 468
**Average Concentration (mg / L)**	6.44 ± 0.30	0.25 ±0.2	6.28 ± 0.11	0.15 ± 0.02	6.89 ± 0.28
**Weight %**	92.1 ± 4.3	3.6 ± 0.3	89.7 ± 1.6	2.1 ± 0.3	98.5 ± 4.0

Copper Nanowires

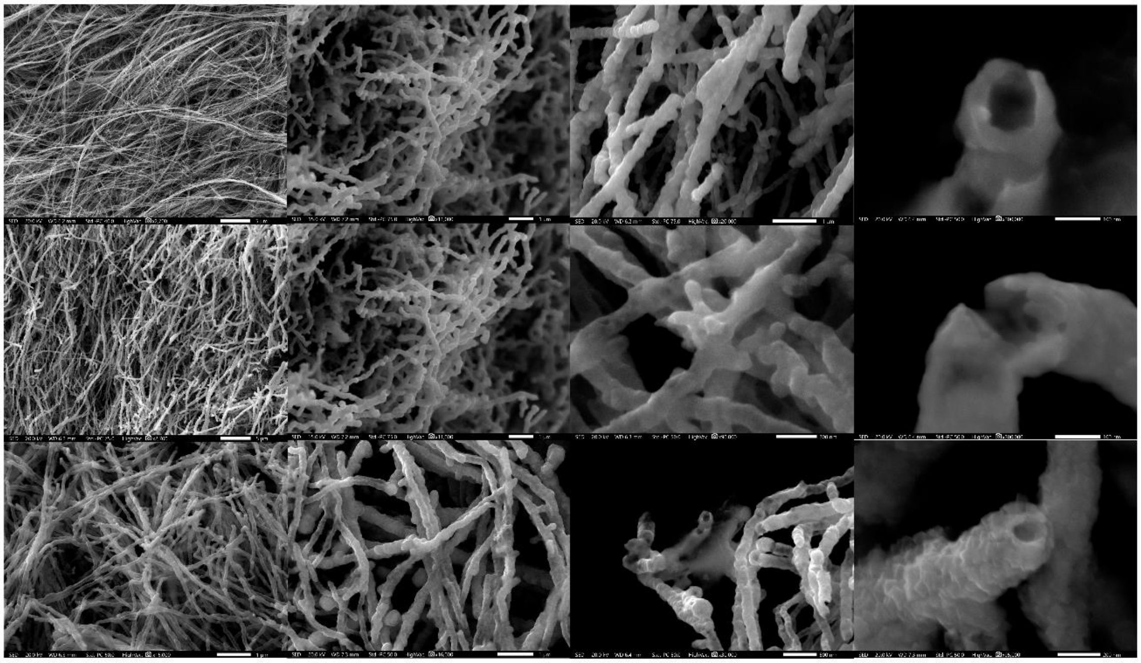

Figure S6. CuNW cross-sections resulting from galvanic displacement of iron nanowires synthesized in a 150 mT field with a 500 mM $CuSO_4 \cdot 5H_2O$ solution.

Platinum Nanotubes

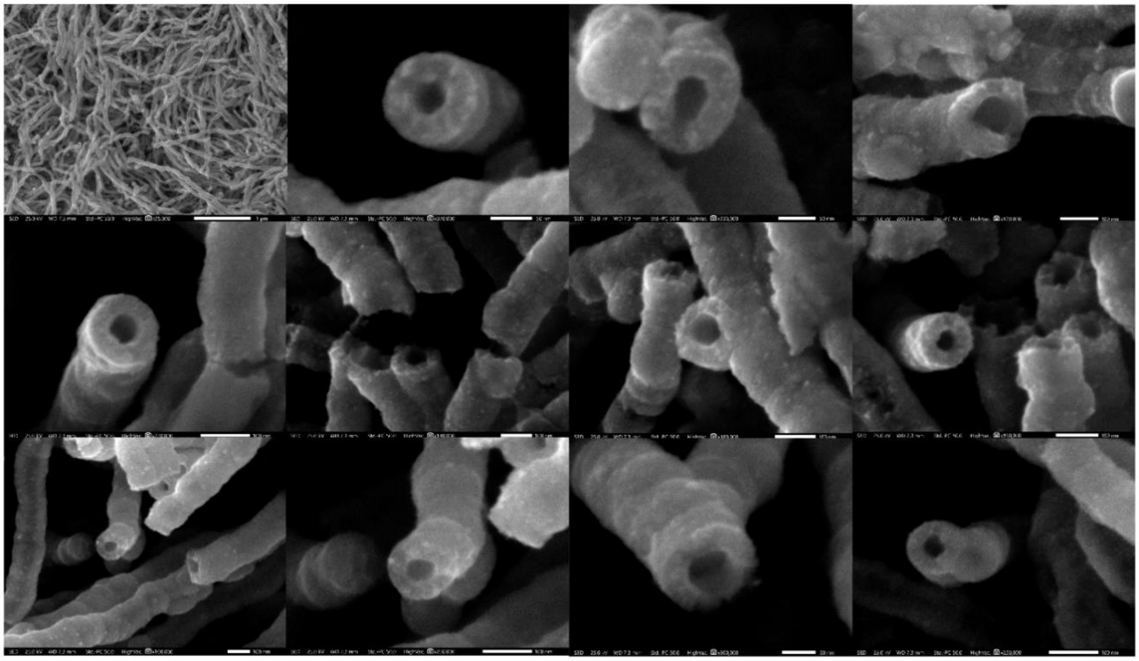

Figure S7. PtNT cross-sections resulting from galvanic displacement of iron nanowires synthesized in a 150 mT field with a 100 mM K_2PtCl_4 solution.

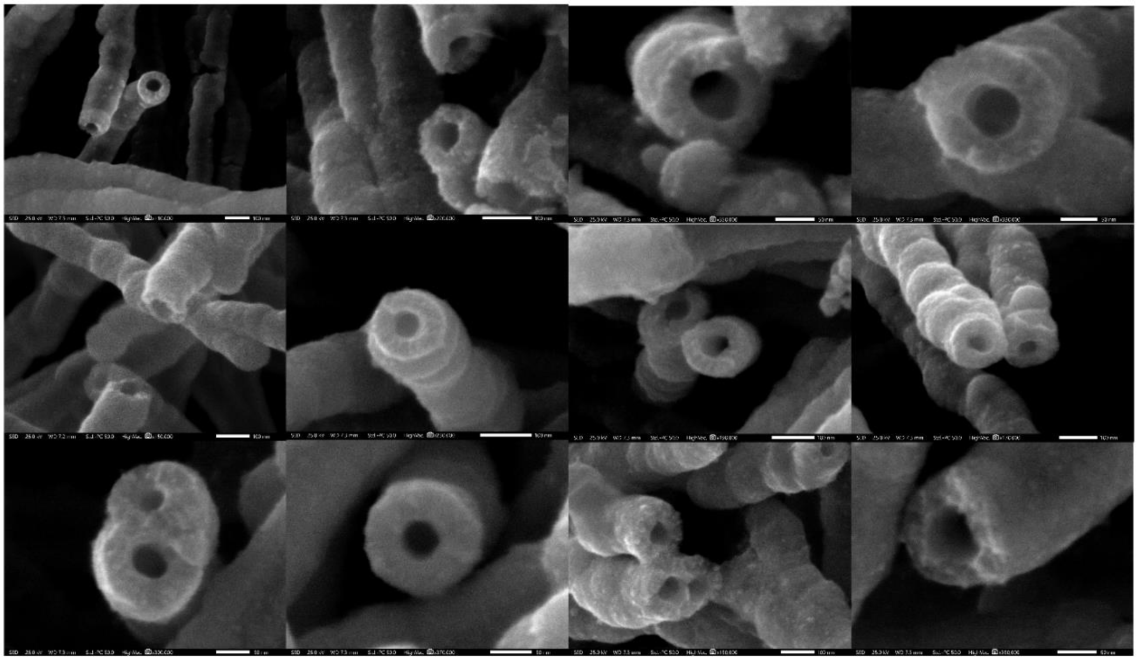

Figure S8. Additional PtNT cross-sections resulting from galvanic displacement of iron nanowires synthesized in a 150 mT field with a 100 mM K_2PtCl_4 solution.

Gold Galvanic Displacement

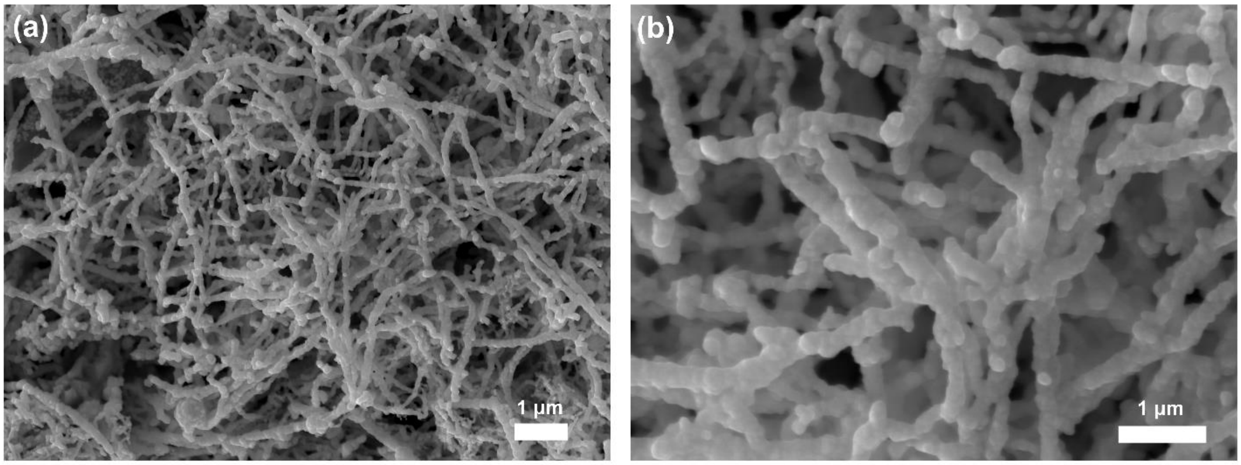

Figure S9. SEM images of gold nanowires prepared from galvanic displacement of the FeNWs with a 0.1 M $HAuCl_4 \cdot 3H_2O$ solution. At 11,000× (a) and 37,000× (b) magnification.

Geometric Analysis of Galvanic Displacement

To assess the galvanic displacement of iron nanowires by copper and platinum ions and to predict the thickness of a resulting nanotube, a geometric model was used with the values shown in Table S5. The geometric model assumes an idealized iron nanowire 1000 nm in length and 95 nm in diameter corresponding to the average nanowire diameter for FeNWs synthesized in a 150 mT field shown in Figure 3a. The iron mass was determined using the density of bulk iron which determined the number of moles of electrons available to facilitate the galvanic displacement of Cu^{2+} and $[PtCl_4]^{2-}$ ions (Pt^{2+}). Complete 100% galvanic displacement efficiency was assumed for a 2 electron transfer to Cu^{2+} and 3 electron transfer from each atom of iron to Pt^{2+} ions (3 mol e^- from Fe(s) : 2 mol e^- to Pt^{2+}). The resulting mass of Cu(s) and Pt(s) was assumed to form a nanotube with inner diameters of 95 nm and 58 nm, respectively based on image analysis shown in Figure 3. The calculated outer diameters for Cu and Pt nanotubes are 134 nm and 144 nm, respectively. The geometrically predicted Cu outer diameter is less than the measured average outer diameter of 228 nm shown in Figure 3(i). The larger observed Cu outer diameter is attributed to a rapid galvanic displacement of Fe by copper ions resulting in dissolution of Fe nanowires transferring electrons to Cu^{2+} along shorter segment lengths of the Fe nanowires. These shorter segment lengths and rounded end caps of Cu nanotubes are seen in Figure 3(c)-(d) and Figure S6. The geometrically predicted Pt nanotube outer diameter of 144 nm is close to the observed average outer diameter of 138 nm. In the case of Pt nanotubes, there is a predominantly outward galvanic displacement growth mechanism from the original Fe nanowire; however, the observed inner diameter in Figure 3(j) is less than the average Fe nanowire diameter of 95 nm, suggesting a partial inward growth mechanism.

Table S5. Galvanic displacement geometric analysis.

Variables	Value	Units
**FeNW Diameter**	95	nm
**FeNW Length**	1000	nm
**FeNW Volume (nm^3)**	7.1E+06	nm^3
**FeNW Volume (cm^3)**	7.1E-15	cm^3
**Fe Density**	7.874	g/cm^3
**Cu Density**	8.96	g/cm^3
**Pt Density**	21.45	g/cm^3
**Molar Mass Fe**	55.845	g/mol
**Molar Mass Cu**	63.546	g/mol
**Molar Mass Pt**	195.084	g/mol
**$Fe^{2+} \leftrightarrow Cu^{2+}$**	1	mol Cu:mol Fe
**$Fe^{2+/3+} \leftrightarrow Pt^{2+}$**	1.5	mole Pt: mol Fe
**Mass Fe**	5.6E-14	g
**Mass Cu (Fe→Cu)**	6.4E-14	g_{Cu}
**Mass Pt (Fe→Pt)**	2.9E-13	g_{Pt}
**Volume Cu**	7.1E-15	cm^3
**Volume Pt**	1.4E-14	cm^3
**Diameter_Inner_Cu**	95	nm
**Diameter_Inner_Pt**	58	nm
**Diameter_Outer_Cu**	134	nm
**Diameter_Outer_Pt**	144	nm

X-ray diffraction (XRD) analysis

XRD peak analysis was done using HighScore (Malvern Panalytical) software. Scans were background subtracted and peak fit and then peaks were assigned to the PDF-2 reference pattern that gave the best match. For samples with multiple crystallite phases, a quantification of each phase was calculated based on the overall peak intensities.

Crystallite sizes for the FeNWs prepared at various applied magnetic field strengths and the galvanically displaced CuNWs and PtNWs was determined according to the Scherrer equation $D = K\lambda/Bcos(\theta)$ where D is the crystallite size, K is the crystallite shape factor (0.89 assuming a spherical crystallite shape), θ is the Bragg diffraction angle, λ = the X-ray wavelength (1.5406 Å for a $Cu_{k\alpha}$ X-ray source) , and B is the sample broadening ($B = B_{observed} - B_{standard}$). A LaB_6 reference standard (NIST standard reference material 660) was used to determine instrumental broadening.

Table S6. Crystallite sizes for the FeNWs prepared at various applied magnetic field strengths

Sample	B observed [°2θ]	B standard [°2θ]	Peak position [°2θ]	B sample [°2θ]	Crystallite size [Å]
**0mT**	0.696	0.192	44.747	0.504	170
**9 mT**	0.722	0.192	44.771	0.53	162
**19 mT**	0.661	0.192	44.725	0.468	183
**37 mT**	0.668	0.192	44.707	0.476	181
**75 mT**	0.655	0.192	44.682	0.463	185
**150 mT**	0.7	0.192	44.672	0.508	169

Table S7. Electron diffraction d-spacings determined from small area electron diffraction. Reference d-spacings were sourced from The Materials Project.[4]

Sample	Electron diffraction d-spacing	Reference d-spacing	Reference
**FeNWs**	1.28	1.29	MP-150 cubic Fe
**FeNWs**	1.25	NA	NA
**FeNWs**	1.03	1.05	MP-150 cubic Fe
**FeNWs**	0.95	.91	MP-150 cubic Fe
**CuNWs**	1.54	1.50	MP-989782 Hexagonal Cu
**CuNWs**	1.24	1.26	MP-30 Cubic Cu
**CuNWs**	1.05	1.08	MP-30 Cubic Cu or MP-989782 Hexagonal Cu
**CuNWs**	1.037	1.033	MP-30 Cubic Cu
**CuNWs**	0.9	0.89	MP-30 Cubic Cu
**PtNTs**	1.17	1.19	MP-126 Cubic Pt
**PtNTs**	1.13	1.138	MP-126 Cubic Pt
**PtNTs**	1.09	0.99	MP-126 Cubic Pt
**PtNTs**	0.68	0.7	MP-126 Cubic Pt
**PtNTs**	0.6	0.67	MP-126 Cubic Pt

X-ray photoelectron spectroscopy (XPS) analysis and fit details

Table S8. Survey scan atomic percentages

Name	Peak Binding Energy (eV)	FeNW Atomic %	CuNW Atomic %	PtNT Atomic %
**O1s**	531	56.07	39.51	16.09
**C1s**	285	24.29	31.27	26.95
**Fe2p**	712	19.64	-	8.45
**Cu2p3**	933	-	24.89	-
**Pt4f**	72	-	-	46.45
**Cl2p**	199	-	1.13	2.05
**Si2p**	102	-	3.21	-

Table S9. Fe2p deconvoluted peak parameters for the FeNWs

Peak Binding Energy (eV)	C1s Corrected Binding Energy (eV)	FWHM (eV)	Area (P) CPS.eV	Atomic Percent (%)	Assignment
**707.08**	707.07	1.48	3295.6	0.14	Fe^0
**708.65**	708.64	1.3	6987.61	0.3	Fe^{2+}
**710.25**	710.24	1.49	71453.96	3.12	Fe^{3+}
**711.27**	711.26	1.74	77328.76	3.38	Fe^{3+}
**712.44**	712.43	2.61	92928.36	4.07	Fe^{3+}
**714.4**	714.39	1.72	7551.64	0.33	Fe^{3+} satellite

Table S10. Cu2p deconvoluted peak fit parameters for the CuNWs

Peak Binding Energy (eV)	C1s Corrected Binding Energy (eV)	FWHM (eV)	Area (P) CPS.eV	Atomic Percent (%)	Assignment
**932.38**	932.37	1.12	111736.2	0.49	Cu^0/Cu^+
**933.79**	933.78	3.37	217538.4	0.95	Cu^{2+}
**941.22**	941.21	2.79	58736.32	0.26	Cu^{2+} satellite
**943.78**	943.77	2.05	42588.47	0.19	Cu^{2+} satellite

Table S11. Pt4f deconvoluted peak fit parameters for the PtNTs

Peak Binding Energy (eV)	C1s Corrected Binding Energy (eV)	FWHM (eV)	Area (P) CPS.eV	Atomic Percent (%)	Assignment
**71.15**	71.76	1	269474.5	24.82	Pt^0 ($4f_{7/2}$)
**71.73**	72.34	2.57	237792	21.91	Pt^{2+} ($4f_{7/2}$)
**74.48**	75.09	0.99	212362.6	-	Pt^0 ($4f_{5/2}$)
**75.11**	75.72	2.57	187394.8	-	Pt^{2+} ($4f_{5/2}$)

Vibrating Sample Magnetometry (VSM)

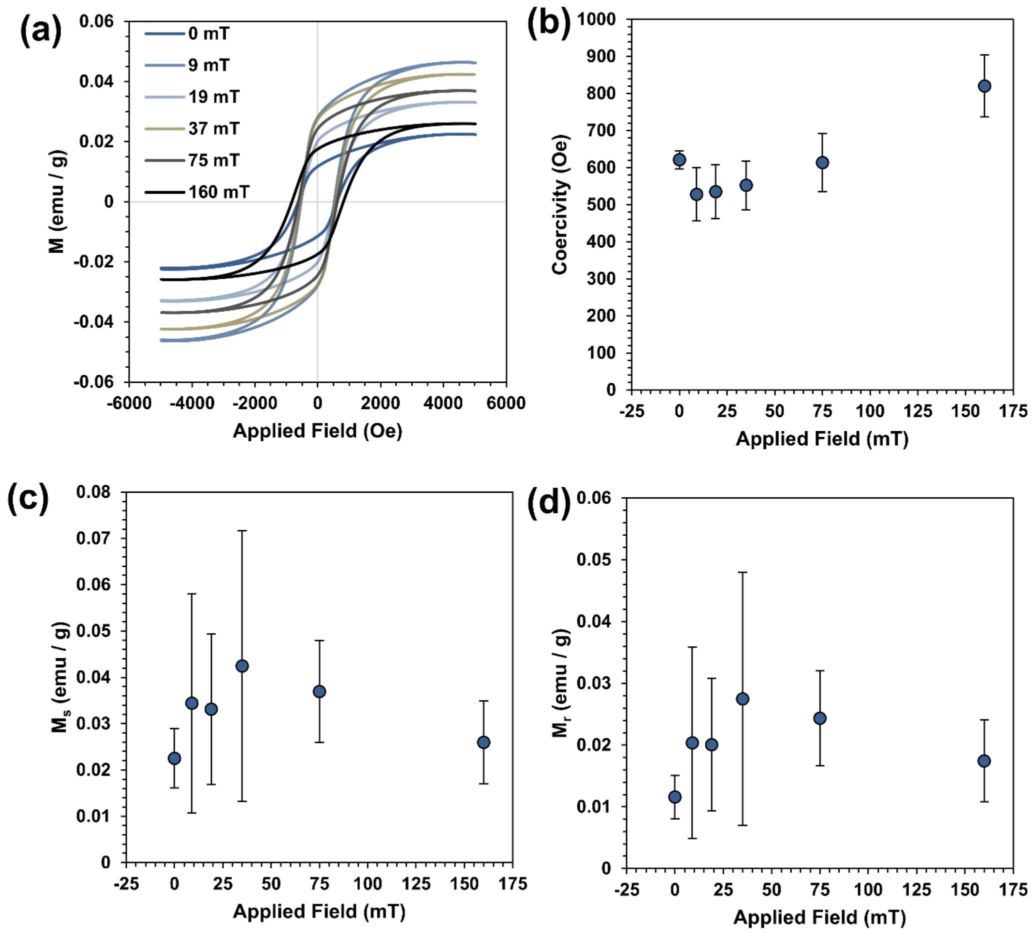

Figure S10. Vibrating sample magnetometry (VSM) measurements for the FeNWs prepared at various magnetic field strengths (a), Coercivity as a function of applied field (b), saturation magnetization as a function of applied magnetic field (c) and remnant magnetization as a function of applied magnetic field (d).

Electrochemical Impedance Spectroscopy Fitting

EIS Fitting. A transmission line model was applied to EIS spectra for CuNWs and PtNTs and fit using Bio-Logic's EC-Lab Zfit module (v.11.10). Initial parameters were estimated using Randomize+Simplex, followed by optimization with Simplex.

Table S 12. Fit values for the transmission line model fits for the CuNWs and PtNTs. The equivalent circuit for the CuNWs was R1+Q1/(R2+Q2/(R3+Q4/(R4+Wd1))) and R1+Q1/(R2+Q2/(R3+Q4/(R4+C1))) for the PtNTs.

Value	CuNWs	Value	PtNTs
R_1 (Ohm)	8.069	R_1 (Ohm)	3.8
Q_1 (F.s^(a - 1))	20.59e-6	Q_1 (F.s^(a - 1))	0.55e-3
a_1	0.825 8	a_1	0.6
R_2 (Ohm)	299.2	R_2 (Ohm)	2.5
Q_2 (F.s^(a - 1))	0.347 5e-3	Q_2 (F.s^(a - 1))	1.5e-3
a_2	0.271 9	a_2	0.9065
R_3 (Ohm)	-100.4	R_3 (Ohm)	15910
Q_4 (F.s^(a - 1))	0.200 3e-3	Q_4 (F.s^(a - 1))	2.092e-6
a_4	1	a_4	0.85
R_4 (Ohm)	-3.936e6	R_4 (Ohm)	-15 560
Rd_1 (Ohm)	3.94e6	C_1 (F)	3.116e-3
td_1 (s)	-8.08e-3	NA	NA
χ^2/ |Z|	0.1772	χ^2/ |Z|	0.119

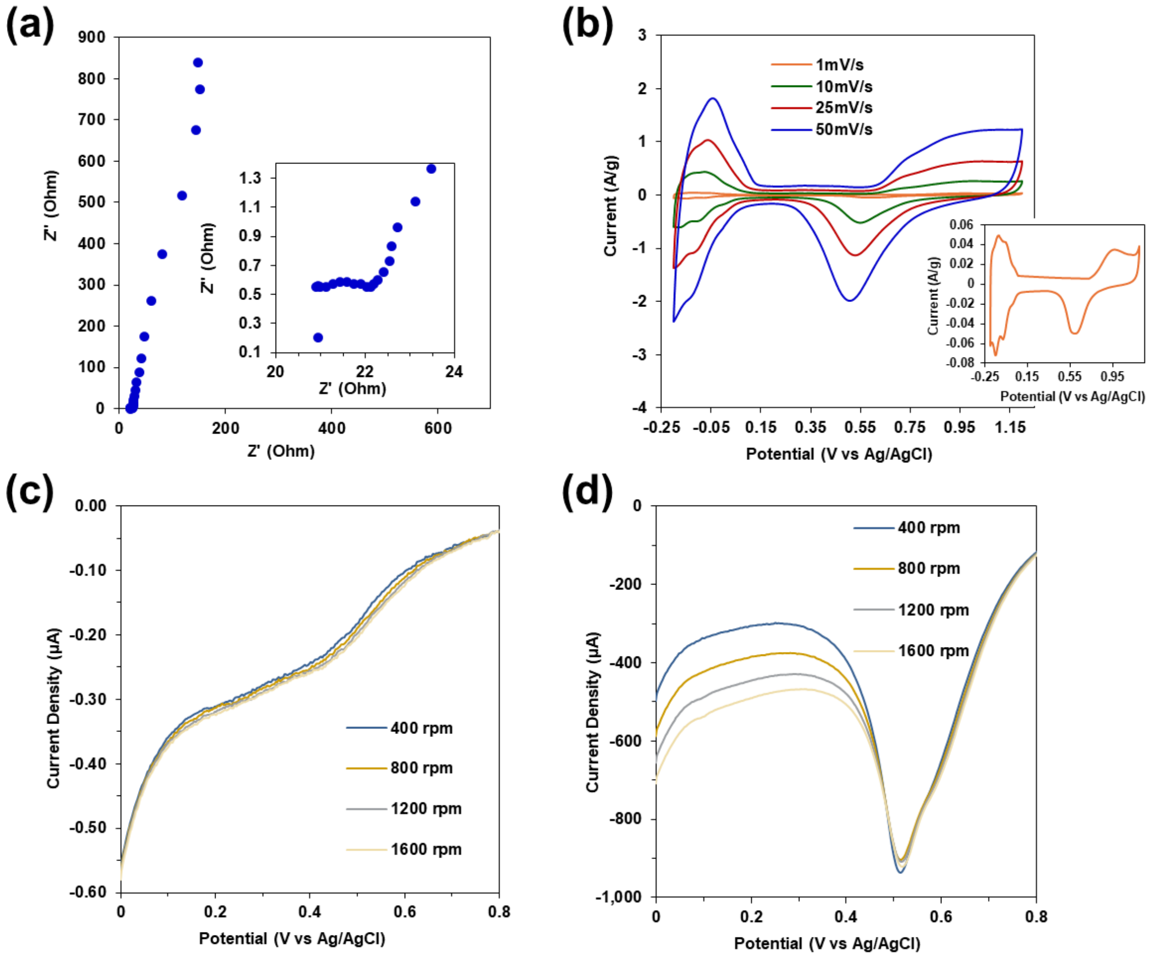

Figure S11. Electrochemical testing in 0.5 M H_2SO_4 of a PtNT film mounted on a GCE with Glassy carbon electrode (GCE) coated with 5 % stock Nafion ambiently dried. Electrochemical impedance spectroscopy (EIS) (a) with high frequency inset, and cyclic voltammetry (b) with 1 mV/s scan inset. Linear sweep voltammetry of a glassy carbon electrode with 5% stock Nafion ambiently dried (c), and a PtNT film mounted on a glassy carbon electrode with 5 µl 5% stock Nafion ambiently dried in oxygen saturated 0.5 M H_2SO_4 electrolyte at a 10 mV/s sweep rate (d).

Linear Sweep Voltammetry

To estimate the number of electrons from the Koutecky-Levich plot in Figure 9i, current densities were converted from A/g to A using the sample mass. For the Koutecky-Levich equation,[5]

$$\frac{1}{i} = \frac{1}{i_o} + \frac{1}{B_L \omega^{1/2}}$$, where $B_L = 0.62nFAD^{2/3}\nu^{-1/6}C$

n is the number of electrons; F is Faraday's constant of 96485 C/mol; A is the area of the electrode with a value of 0.20 cm^2; D is the diffusion coefficient of oxygen with a value of 1.4 x 10^{-5} cm^2/s; ν is the kinematic viscosity with a value of 0.010 cm^2/s; and C is the concentration of oxygen, assumed to be. 1.1 x 10^{-6} mol/cm^3.

Video S1 – Fe aerogel chemical reduction.

Video S2 – Fe aerogel mechanical compression.

Supporting References

(1) Schneider, C. A.; Rasband, W. S.; Eliceiri, K. W. NIH Image to ImageJ: 25 Years of Image Analysis. *Nature Methods* **2012**, *9* (7), 671–675. https://doi.org/10.1038/nmeth.2089.

(2) Püspöki, Z.; Storath, M.; Sage, D.; Unser, M. Transforms and Operators for Directional Bioimage Analysis: A Survey. In *Focus on Bio-Image Informatics*; De Vos, W. H., Munck, S., Timmermans, J.-P., Eds.; Springer International Publishing: Cham, 2016; pp 69–93. https://doi.org/10.1007/978-3-319-28549-8_3.

(3) Persson, N. E.; McBride, M. A.; Grover, M. A.; Reichmanis, E. Automated Analysis of Orientational Order in Images of Fibrillar Materials. *Chem. Mater.* **2017**, *29* (1), 3–14. https://doi.org/10.1021/acs.chemmater.6b01825.

(4) Jain, A.; Ong, S. P.; Hautier, G.; Chen, W.; Richards, W. D.; Dacek, S.; Cholia, S.; Gunter, D.; Skinner, D.; Ceder, G.; Persson, K. A. Commentary: The Materials Project: A Materials Genome Approach to Accelerating Materials Innovation. *APL Materials* **2013**, *1* (1), 011002. https://doi.org/10.1063/1.4812323.

(5) Bard, A. J.; Faulkner, L. R.; White, H. S. *Electrochemical Methods: Fundamentals and Applications*; John Wiley & Sons, 2022.
